# Topoisomerase 1 facilitates nucleosome reassembly at stress genes during recovery

**DOI:** 10.1093/nar/gkad1066

**Published:** 2023-11-13

**Authors:** Montserrat Vega, Rubén Barrios, Rodrigo Fraile, Kevin de Castro Cogle, David Castillo, Roger Anglada, Ferran Casals, José Ayté, Ernesto Lowy-Gallego, Elena Hidalgo

**Affiliations:** Oxidative Stress and Cell Cycle Group, Universitat Pompeu Fabra, Barcelona 08003, Spain; Oxidative Stress and Cell Cycle Group, Universitat Pompeu Fabra, Barcelona 08003, Spain; Oxidative Stress and Cell Cycle Group, Universitat Pompeu Fabra, Barcelona 08003, Spain; BatchX Inc., San Jose, CAUSA; BatchX Inc., San Jose, CAUSA; Genomics Core Facility, Universitat Pompeu Fabra, Barcelona 08003, Spain; Genomics Core Facility, Universitat Pompeu Fabra, Barcelona 08003, Spain; Oxidative Stress and Cell Cycle Group, Universitat Pompeu Fabra, Barcelona 08003, Spain; European Molecular Biology Laboratory, European Bioinformatics Institute, Wellcome Genome Campus, Hinxton, Cambridge CB10 1SD, UK; Oxidative Stress and Cell Cycle Group, Universitat Pompeu Fabra, Barcelona 08003, Spain

## Abstract

Chromatin remodeling is essential to allow full development of alternative gene expression programs in response to environmental changes. In fission yeast, oxidative stress triggers massive transcriptional changes including the activation of hundreds of genes, with the participation of histone modifying complexes and chromatin remodelers. DNA transcription is associated to alterations in DNA topology, and DNA topoisomerases facilitate elongation along gene bodies. Here, we test whether the DNA topoisomerase Top1 participates in the RNA polymerase II-dependent activation of the cellular response to oxidative stress. Cells lacking Top1 are resistant to H_2_O_2_ stress. The transcriptome of Δ*top1* strain was not greatly affected in the absence of stress, but activation of the anti-stress gene expression program was more sustained than in wild-type cells. Top1 associated to stress open reading frames. While the nucleosomes of stress genes are partially and transiently evicted during stress, the chromatin configuration remains open for longer times in cells lacking Top1, facilitating RNA polymerase II progression. We propose that, by removing DNA tension arising from transcription, Top1 facilitates nucleosome reassembly and works in synergy with the chromatin remodeler Hrp1 as opposing forces to transcription and to Snf22 / Hrp3 opening remodelers.

## Graphical Abstract

**Graphical Abstract:**
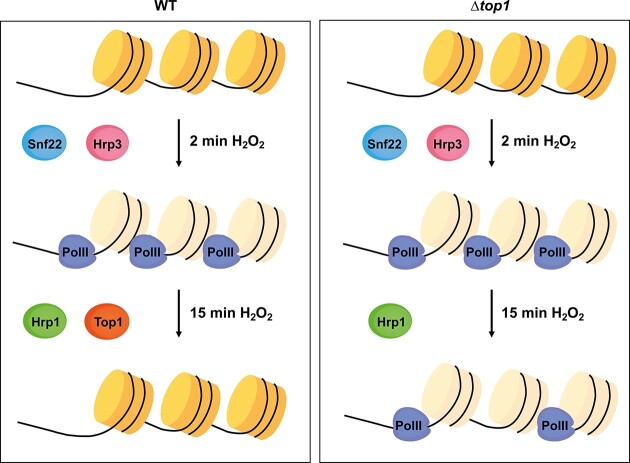

## Introduction

Cells have to adapt to changing environments. In eukaryotic cells, mitogen-activated protein (MAP) kinase pathways are key elements in the response to environmental signals, and they regulate common anti-stress gene expression programs to allow cell adaptation (1). Phosphorylation of transcription factors by MAP kinases under stress conditions is essential for their activation at the cell nucleus (2), where they promote wide changes in gene expression patterns. Most of these changes are transcriptional.

In fission yeast *Schizosaccharomyces pombe*, the Sty1/Spc1 MAP kinase is activated by phosphorylation upon several stresses, promoting its nuclear accumulation into the nucleus. Once there, Sty1 phosphorylates and activates the basic zipper (bZIP)-containing transcription factor Atf1 to promote transcriptional activation or repression of genes (3–6), although not all these transcriptional changes depend fully on Atf1 (7,8). In particular, in response to hydrogen peroxide (H_2_O_2_) more than 550 genes are up-regulated >2-fold in a Sty1-dependent manner (9). The lack of Sty1 or of Atf1 makes cells sensitive to the addition of extracellular peroxides (10–12). Therefore, controlling the shift of the cellular transcriptional program is essential to promote survival in front of harsh conditions.

In order to promote these dramatic transcriptomic changes, stress-activated transcription factors such as Atf1 have to trigger chromatin remodeling. Eukaryotic DNA is wrapped around the histone core (2 moieties of histones H2A, H2B, H3 and H4) forming nucleosomes, which can be packed around each other to different extents. Fission yeast lacks the linker histone H1, and *in vitro* reconstituted nucleosomal arrays form thinner fibers than those of human cells (13). Most organisms, and fission yeast is not an exception, pack their DNA in regularly spaced nucleosomes (14). Many coding genes have a nucleosome depleted region (NDR) located upstream of the transcription start site (TSS), and the stress genes controlled by Sty1 and Atf1 display a wider than usual NDR at their promoters, 400-bp long, prior to stress activation (15). The proper localization and organization of the open reading frame (ORF) nucleosomes at stress genes is at least in part dependent on Atf1 even prior to stress (16). Stress-dependent gene activation transiently changes the chromatin architecture at the gene bodies, with partial eviction of the nucleosomes located downstream of the TSS, to allow RNA polymerase II (Pol II) elongation (15). Activated transcription factors and RNA polymerases cause alteration of the nucleosome landscapes by recruiting one or several of the following types of proteins: histone modifiers, chromatin remodelers or DNA topoisomerases.

Transcription regulation of stress genes by activated Atf1 has been linked to the acetylation of histones by the Gcn5 acetyl transferase of the SAGA complex, not only at stress genes but also at *ade6-M26* and *fbp1* (15,17,18). In a genome-wide screen, mutants lacking components of complexes promoting robust transcription such as Paf1 complex [which couples histone modifications to efficient transcription elongation; for a review, see (19)], were isolated as sensitive to oxidative stress (20). Whether the activated transcription factor or elongating Pol II are recruiting these histone modifying complexes is still under study.

Chromatin remodelers use energy from ATP to promote the eviction or sliding of nucleosomes and favor transcription elongation, among other functions. Even though direct connections between chromatin remodelers and full induction of stress genes have not yet been established in fission yeast, some of these remodelers have been connected to other nuclear functions of Atf1. Thus, the absence of Snf22 or Hrp3 causes a marked reduction of Atf1-dependent meiotic recombination at the *ade6-M26* locus (17,21), and compromises Atf1- and glucose depletion-dependent activation of *fbp1* (18).

Since DNA topology can also affect nucleosome stability and chromatin architecture *in vitro* (22–24) and *in vivo* (25), DNA topoisomerases may also contribute to the regulation of the stress gene expression program. These enzymes disentangle the topological problems that arise in double-stranded DNA; thus, when strand unwinding occurs during transcription and replication, DNA topoisomerases compensate with the transient introduction of breaks in DNA. Regarding transcription, RNA polymerases generate positive supercoils in front of the advancing transcription complex and negative supercoils behind, according to the twin supercoiled model proposed by Wang (26).

In order to remove DNA tension, eukaryotic cells express two types of DNA topoisomerases, which induce either single-strand (type I) or double-strand (type II) DNA breaks [for reviews, see (27–29)]. In general, type II DNA topoisomerases, being potentially very dangerous enzymes, are required to segregate replicated chromosomes, among other functions, and they are often essential in most eukaryotic models [for a review, see (30)]. On the contrary, type I enzymes are often dispensable, and have been linked to transcription. It has been suggested that high transcription rates at specific genes cause increased type I topoisomerase recruitment, probably through the interaction of these DNA topoisomerases with elongating Pol II, to favor efficient transcription (31,32).

Type I enzymes resolve torsional stress by introducing a single-strand break and covalently binding to the 3′-end of the nick, to generate a transient covalent protein-DNA intermediate called Top1cc, which can then rotate around the intact DNA complementary strand prior to re-ligation of the single strand nick. During this process, mutations can arise as a consequence of the cleavage and binding reaction, for instance in the presence of the Top1 poison camptothecin (CPT) [for a review, see (33)]. The accumulation or persistence of Top1cc adducts is counteracted by several repair factors including tyrosyl-DNA phosphodiesterase (Tdp1); at least in fission yeast, Tdp1 also processes Top1-independent lesions arising from oxidative stress in quiescent fission yeast (34) or upon chromate damage to DNA (35).

As suggested above, DNA topology may affect nucleosome positioning at transcribed genes. In budding yeast, it has been recently reported that Top2 accumulates at gene boundaries, where negative supercoils accumulate. On the contrary, Top1 accumulates at gene bodies, together with positive supercoils and with Pol II; accumulation of Pol II and Top1 mirror the levels of expression, being high in highly expressed genes (25). These results suggest that Pol II progression might strongly rely on Top1 for resolving the topological stress which accumulates in front of the transcription machinery.

Regarding fission yeast's DNA topoisomerases, while Top2 is essential Top1 is not, likely because Top2 (type II) can substitute for Top1 function (36). The group of Ekwall proposed that Top1 activity is directly required for efficient nucleosome disassembly at gene promoter regions, and in its absence nucleosome occupancy increases at 5′ regions and transcription is reduced (37).

In a genome-wide screen, we demonstrated that hundreds of genes are required to coordinate the transcription of stress genes for the acquisition of tolerance to oxidative stress, and that defects in either histone modifiers or chromatin remodeler complexes jeopardize wild-type tolerance to peroxides (20). Regarding DNA topology, we have here tested whether deletion of the non-essential *top1* gene could sensitize cells to H_2_O_2_ stress. On the contrary, cells lacking Top1 are unambiguously resistant to oxidative stress. RNA sequencing analysis demonstrates that stress genes are induced at a higher extent in Δ*top1* than in wild-type cells. As determined genome-wide, nucleosomal DNA sequencing demonstrates that while in wild-type cells the nucleosomes at stress gene bodies return to the starting positions short after stress imposition, cells lacking Top1 display longer kinetics of chromatin closing and nucleosome re-assembly. Our study suggests that Top1 contributes to resolving DNA tension ahead of Pol II, but in its absence positive supercoils may impair nucleosome repositioning after the first rounds of transcription, facilitating further Pol II elongation steps.

## Materials and methods

### Yeast strains, plasmids and growth conditions

Cells were grown in rich medium (YE5S) as described previously (38). All yeast strains used in this study are outlined in Supplementary Table S1 and were constructed by standard genetic methods. To generate a strain expressing a catalytically-dead Top1.Y773F, we used *SpEDIT*, a CRISPR/Cas9 method (39), using the following primers (see Table S2): primers OLEH-AC41 and OLEH-AC42 were used for sgRNA cloning, and primers OLEH-AC43 and OLEH-AC44 for generating the homologous recombination fragment including the mutation.

### H_2_O_2_ survival assays

Assays were performed as previously described (40). Briefly, strains were grown at 30°C in YE5S until they reached logarithmic phase, at an OD_600_∼0.5. The same number of cells (10^5^ and 1/10 serial dilutions) in 3 μl was spotted on YE5S agar plates with or without the indicated concentration of H_2_O_2_. The spots were allowed to dry and the plates were incubated at 30°C for 2–4 days.

### TCA extracts and immunoblot analysis

Yeast cells were grown in YE5S until an OD_600_ of 0.5 was reached. Trichloroacetic acid (TCA) was added to a final concentration of 10%. Cells were pelleted and washed in 20% TCA. The pellets were resuspended in 100 μl of 12.5% TCA and lysed by vortexing in the presence of glass beads. Cell lysates were pelleted, washed in acetone, and dried. Pellets were resuspended in Tris buffer (0.1 M Tris–HCl pH 8.0, 1 mM EDTA, 1% SDS), loading buffer was added and samples were boiled for 5 min at 100°C. Samples were separated by SDS-PAGE and detected by immunoblotting with polyclonal anti-Atf1 (40), monoclonal anti-GFP (Takara; 632381), or monoclonal anti-Tubulin (SIGMA; T5168).

### RNA isolation and analysis by northern blot

Cells grown in YE5S until an OD_600_ of 0.5 were left either untreated or were treated for 30 min with 1 mM of H_2_O_2_. 25 ml of yeast cultures were then centrifuged at 1500 rpm for 3 min and washed with H_2_O, and cell pellets were immediately frozen in ice. Each sample was then resuspended in 0.4 ml of AE buffer (50 mM sodium acetate, pH 5.3, 10 mM EDTA, pH 8.0). Sodium dodecyl sulfate was then added to a final concentration of 1%, and proteins and DNA were extracted by adding 0.4 ml of phenol-chloroform and incubated at 65°C for 5 min. The aqueous phase was separated by centrifugation at 10 000 × g for 2 min at 4°C. After chloroform extraction, RNA was precipitated with ethanol. Extracted RNA was loaded on formaldehyde agarose gels and transferred to membranes, which were hybridized with the [α-^32^P]-dCTP labeled *ctt1* or *srx1*, containing the complete ORF.

### RNA analysis by quantitative PCR

Reverse transcription was performed with purified RNA treated with DNase I using Reverse Transcription System of Applied Biosystems (Thermo Fisher Scientific), following the manufacturer's instructions. The cDNA was quantified by real-time quantitative PCR on Light Cycler II using Light Cycler 480 SYBR Green I Master (Roche). The error bars (SD) were calculated from three biological replicates, as indicated, and *act1* gene was used as a control for normalization. Fold induction was calculated comparing the value of each strain and condition to that of the untreated conditions of the wild-type strain. Primers used are included in Supplementary Table S2.

### Chromatin immuno-precipitation (ChIP)

Cells were grown in YE5S medium and chromatin isolation and immunoprecipitation were carried out as previously described (15), with minor modifications. Briefly, cells from 50 ml of culture were cross-linked with 1% formaldehyde (directly added to cultures) for 10–20 min. After stopping cross-linking with 125 mM glycine, pellets were lysed with a bead beater, and lysates were sonicated, yielding chromatin fragments of ∼400 bp average size. Chromatin was immuno-precipitated with specific antibody [5 μl of anti-HA antiserum (12CA5; house-made), 1 μl of anti-Myc polyclonal (SIGMA; C3956), 1 μl of anti-V5 monoclonal (BioRad; MCA1360), 1 μl anti-phospho-Ser2 RNA Pol II CTD polyclonal (Abcam; ab5095)], overnight at 4°C rotating. Beads were washed, DNA was eluted and formaldehyde cross-linking was reversed. After protein digestion and chromatin extraction, DNA was amplified by quantitative real-time quantitative PCR using Light Cycler 480 SYBR Green I Master (Roche). The error bars (SD) were calculated from two or three biological replicates, as indicated, and primers from an intergenic region or a mitochondrial DNA (*mtDNA*) region were used as controls. Primers used are included in Supplementary Table S2.

### RNA sequencing and analysis

Total RNA from *S. pombe* YE5S cultures, treated or not for 30 min with 1 mM H_2_O_2_, was obtained and processed as described above. As suggested by ENCODE guidelines for RNA sequencing experiments, two biological replicates were performed and analyzed. Libraries were prepared using the TruSeq Stranded mRNA Sample Prep Kit v2 (ref. RS-122–2101/2) following manufacturer's instructions. Briefly, 1 μg of total RNA was used for poly (A)-mRNA selection using streptavidin-coated magnetic beads and were subsequently fragmented to approximately 300 bp. cDNA was synthesized using reverse transcriptase (SuperScript II, ref. 18064–014, Invitrogen) and random primers. The second strand of the cDNA incorporated dUTP in place of dTTP. Double-stranded DNA was further used for library preparation. dsDNA was subjected to A-tailing and ligation of the barcoded Truseq adapters. All purification steps were performed using AMPure XP beads. Library amplification was performed by PCR from the purified library using the primer cocktail supplied in the kit. Final libraries were analyzed using Agilent DNA 1000 chip to estimate the quantity and check size distribution, and were then quantified by quantitative PCR using the KAPA Library Quantification Kit (ref. KK4835, KapaBiosystems) prior to amplification with Illumina's cBot. Sequencing was done using the HiSeq2000, Single Read, and 50 nts (v3). For the RNAseq analysis, raw FASTQ files were first evaluated using quality control checks from FastQC 0.11.9. Expression was estimated for each sample using Salmon 1.2.1, performing a quasi-mapping read alignment against *S. pombe* ASM294v2 transcriptome. The correlation between biological duplicates for all RNA-seq couples according to Pearson coefficient was > 0.98. Quantification results were then imported to DESeq2 1.24.0 for differential expression analysis. The threshold adjusted p-value used to identify differentially expressed genes was 0.1. Heatmaps were obtained using the function pheatmap (1.0.12) from R package.

### Mononucleosome purification and sequencing

Cells fixed with formaldehyde directly added to cultures were subjected to chromatin isolation and MNase digestion as described before (16). As suggested by ENCODE guidelines for nuclease sequencing experiments, two biological replicates were performed and analyzed. For each biological replicate of the nucleosomal map to be determined, mononucleosomal DNA fragments from two independent cell cultures were purified from 2% agarose gels run in Tris-acetic acid-EDTA (TAE), DNA was extracted with Quantum Prep Freeze’N Squeeze DNA gel extraction spin columns (Bio-Rad). DNA libraries were prepared with Roche Kapa Hyper Plus, following manufacturer instructions, and subjected to paired-end sequencing using Illumina Nextseq 500 platform. Short reads produced in this work were aligned against the genome of *S. pombe* 972 using the Bowtie2 software with default parameters (41). The SAM format file produced by Bowtie2 was converted into a BAM file using SAMtools (42). SAMtools was also used to filter low quality alignments (supplementary, improper paired, non-primary alignments and reads with less than 20 of mapping quality). The Picard MarkDuplicates function (https://broadinstitute.github.io/picard/) was then used to remove duplicate reads from the BAM file. The DANPOS2 algorithm (43) was used to remove clonal reads, adjust read length, perform quantile normalization, identify nucleosomes in the genome and calculate differences in nucleosome occupancy. Correlation between biological duplicates was calculated using Pearson coefficient using deepTools.

### Mononucleosome purification and nucleosome scanning analysis

Nucleosome profiles at the *ctt1* gene were determined by nucleosome scanning as previously described (15). Briefly, chromatin from triplicate cell cultures were obtained as explained above and treated with increasing concentrations of MNase to yield mononucleosomes as determined by agarose electrophoresis. Non-gel purified fractions of mononucleosomal DNA were PCR-amplified with pairs of overlapping primers covering ∼0.8 kb of the *ctt1* gene (Supplementary Table S2). Pairs of primers centered at positions, relative to the TSS, of -291 (OLEH-B48 and OLEH-B49), -221(OLEH-B50 and OLEH-B51), -144 (OLEH-B52-OLEH-B53), -77 (OLEH-B54 and OLEH-B55), -43 (OLEH-B56 and OLEH-B57), +45 (OLEH-B58 and OLEH-B59), +137 (OLEH-B60 and OLEH-B61), +238 (OLEH-B62 and OLEH-B63), +323 (OLEH-B64 and OLEH-B65), +415 (OLEH-B66 and OLEH-B67), +498 (OLEH-B68 and OLEH-B69) and + 548 (OLEH-B92 and OLEH-B93).

### Statistical analysis

Unless otherwise stated, all experiments were performed at least three times and representative ones are shown. For statistical analysis two-sided *t*-test was performed.

## Results

### Cells lacking Top1 are resistant to oxidative stress

**Figure 1.**
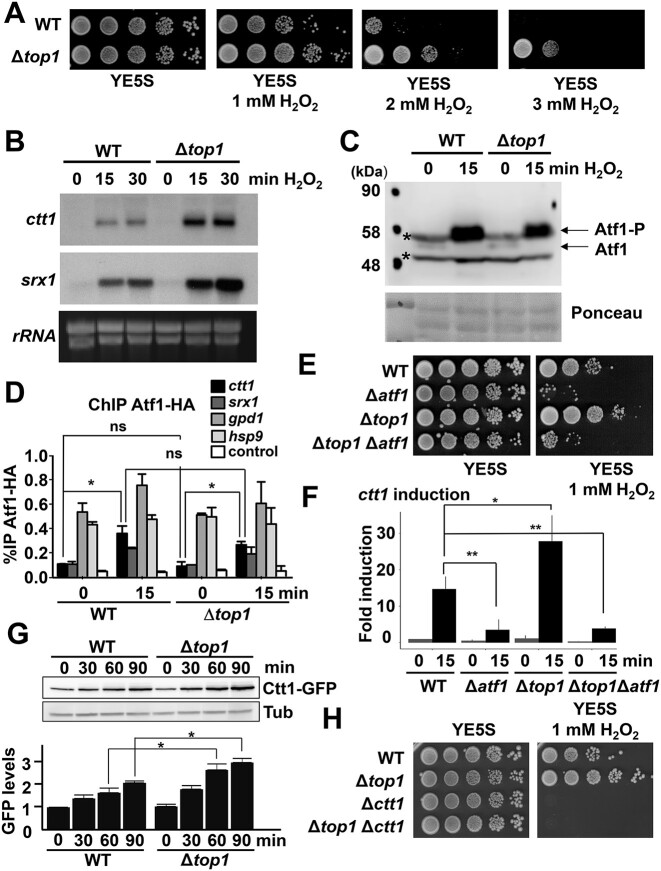
Δ*top1* strains display increased survival to oxidative stress due to enhanced transcription of stress genes. (**A**) Δ*top1* strain is resistant to H_2_O_2_. Serial dilutions of exponentially growing cultures of the strains 972 (WT) and RF14 (Δ*top1*) were spotted in YE5S with or without the indicated concentrations of H_2_O_2_. (**B**) Δ*top1* strain has increased expression of stress response genes. Total RNA from YE5S cultures of the strains as in A, treated or not with 1 mM H_2_O_2_, was obtained and analyzed by northern blot with labeled *ctt1* and *srx1* probes. rRNA was used as loading control. (**C**) Atf1 protein levels and phosphorylation upon stress are not affected in Δ*top1* background. TCA protein extracts from cell cultures of strains as in A, treated or not with 1 mM H_2_O_2_, were prepared and analyzed by western blot using polyclonal antibodies (anti-Atf1). Ponceau staining was used as a loading control. (**D**) Atf1 is recruited normally to stress gene promoters in Δ*top1* cells. YE5S cultures of strains CS38 (*atf1-HA*) and RF76 (Δ*top1 atf1-HA*) were treated or not with 1 mM H_2_O_2_. for 15 min. ChIP experiments using anti-HA antibodies, coupled to quantification by real-time quantitative PCR, were performed using primers covering promoter regions (*ctt1, srx1, gpd1*, and *hsp9*). Primers of an intergenic region were used as control. Each bar represents the mean value and SD from two biological replicates. Significant differences between samples were determined by a two-sided *t*-test (*P< 0.05; ns, non-significant). (**E**) Δ*top1* lose their resistance to H_2_O_2_ when Atf1 is not present. Experiment performed as in A, using strains 972 (WT), MS98 (Δ*atf1*), RF14 (Δ*top1*) and RF52 (Δ*atf1* Δ*top1*). (**F**) Overexpression of *ctt1* in a Δ*top1* background is lost in the absence of Atf1. Expression of *ctt1* was analyzed by quantitative PCR in strains as in E. Amplification with *act1* primers was used as a control for normalization. Each bar represents the mean value and SD from three biological replicates. Significant differences between samples were determined by a two-sided *t*-test (*P< 0.05, **P< 0.01). (**G**) Ctt1-GFP induction in response to oxidative stress. TCA extracts from cell cultures of strains PG102 (WT) and MV119 (Δ*top1*), carrying an endogenous *ctt1-GFP* locus and treated or not with 1 mM H_2_O_2_, were prepared and analyzed by western blot using polyclonal antibodies (anti-GFP). Anti-Tubulin antibody was used as loading control. Bar plot below show quantification of the western blots. Each bar represents the mean value and SD from three biological replicates. Significant differences were determined by a two-sided *t*-test (*P< 0.05). (**H**) Experiment performed as in A, using strains 972 (WT), RF14 (Δ*top1*), EP198 (Δ*ctt1*) and MV117 (Δ*ctt1* Δ*top1*).

With the idea that accumulation of topological stress at the DNA of stress genes may have a negative impact on their transcription and on stress survival, we tested the tolerance to peroxide stress of cells lacking the non-essential Top1. To our surprise, cells lacking Top1 were resistant to H_2_O_2_ on plates, when compared to a wild-type strain (Figure 1A). This phenotype was observed for Top1-deficient cells of different origins, either lacking almost the whole ORF as designed by Bioneer (44) (Δ*top1* in Figure 1A and Supplementary Figure S1AB) or missing only the last 301-bp of the 3′ region coding for the catalytic domain (*top1::ura4*; Supplementary Figure S1A). Cells expressing a catalytically-dead version of the enzyme, Top1.Y773F, also displayed resistance to peroxides (Supplementary Figure S1B), suggesting that lack of topoisomerase activity is involved in this phenotype. In fact, the Top1 deficient strains displayed resistance to camptothecin, as expected (Supplementary Figure S1C): this drug inhibits Top1 and promotes the accumulation of Top1cc adducts, causing DNA damage and cell toxicity in wild-type cells (45). The tyrosyl-DNA phosphodiesterase protein, Tdp1, removes Top1cc adducts together with the Rad16-Swi10 module (46). Nevertheless, the absence of Tdp1 did not have any effect on wild-type tolerance to oxidative stress (Supplementary Figure S1D), suggesting that DNA damage is not involved in the resistance phenotype of cells lacking Top1.

We determined by northern blot that the *ctt1, srx1* and *hsp9* genes, coding for the anti-stress proteins catalase, sulfiredoxin and the chaperone Hsp9, respectively, were up-regulated in Δ*top1* cells to a larger extent that in a wild-type strain upon H_2_O_2_ stress (Figure 1B and Supplementary Figure S1E); the same trend was observed in the *top1::ura4* background (Supplementary Figure S1E), as well as in the Top1.Y773F-expressing strain (Supplementary Figure S1F).

The *ctt1, srx1* and *hsp9* genes are up-regulated upon oxidative stress in a Sty1- and Atf1-dependent manner (9). Nevertheless, we showed by western blot that Atf1 phosphorylation was not exacerbated in cells lacking Top1 (Figure 1C). Similarly, we determined by ChIP that Atf1 recruitment to stress promoters was unaltered in Δ*top1* cells (Figure 1D). However, the stress phenotype of cells lacking Top1 was fully dependent on the presence of Atf1, both regarding resistance to stress (Figure 1E) or activation of the stress *ctt1* gene (Figure 1F).

We previously demonstrated that overexpression of catalase (Ctt1) alone is essential for cell tolerance to oxidative stress, as ectopic expression of only this protein totally or partially suppresses the absence of components of the Sty1 or Pap1 antioxidant cascades (12). As determined by western blot, the levels of Ctt1-GFP protein expressed from its own genomic locus were enhanced by H_2_O_2_ treatment (20), and this over-expression was significantly higher in cells lacking Top1 (Figure 1G). Furthermore, the stress resistance of cells lacking Top1 was fully abolished by Ctt1 depletion (Figure 1H).

### The transcriptome of cells lacking Top1 is enriched in stress transcripts

**Figure 2.**
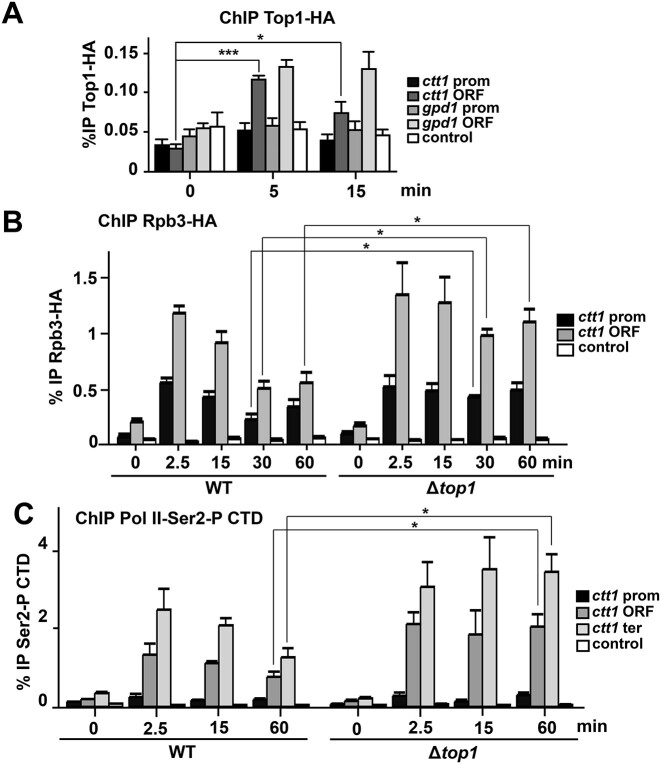
Role of Top1 in Pol II recruitment. (**A**) Top1-HA is recruited to the ORFs of stress genes after stress imposition. ChIP experiments as in Figure 1D using anti-HA antibodies were performed from cultures of strain RF92 (*top1-HA*), using primers covering promoter or ORF regions of *ctt1* and *gpd1*. Primers of an intergenic region were used as control. Each bar represents the mean value and SD from three biological replicates. Significant differences were determined by a two-sided *t*-test (*P< 0.05, ***P< 0.001). (**B**) Stress-dependent recruitment of the Pol II subunit Rpb3-HA to the *ctt1* gene. ChIP experiments as in Figure 1D using anti-HA antibodies were performed from cultures of strains JF5 (*rpb3-HA*) and RF96 (Δ*top1rpb3-HA*), using primers covering promoter or ORF regions of *ctt1*. Primers of a *mtDNA* region were used as control. Each bar represents the mean value and SD from three biological replicates. Significant differences were determined by a two-sided *t*-test (*P< 0.05). (**C**) Recruitment of Pol II phosphorylated at Ser2 of its CTD. ChIP experiments as in Figure 1D using anti-Ser2-P antibodies were performed from cultures of strains 972 (WT) and RF14 (Δ*top1*), using primers covering promoter, ORF or terminator regions of *ctt1* gene. Primers of a *mtDNA* region were used as control. Each bar represents the mean value and SD from three biological replicates. Significant differences were determined by a two-sided *t*-test (*P< 0.05).

We tagged the endogenous Top1 with HA at its C-terminal domain, assayed that cells expressing this tagged version had wild-type tolerance to H_2_O_2_ (Supplementary Figure S2A), and tested by ChIP its localization at promoters and ORFs of stress genes, both before and after stress imposition. As shown in Figure 2A, Top1-HA accumulated at ORFs of the *ctt1* and *gpd1* genes after peroxide stress; we did not detect the chimera at promoters. The catalytically dead Top1.Y773F mutant, also tagged with HA, was recruited to the ORFs of stress genes after stress to the same extent than wild-type Top1 (Supplementary Figure S2B).

Regarding recruitment of the transcriptional machinery, the Rpb3-HA subunit of Pol II was detected at both promoters and ORFs after stress imposition, as previously reported (8) (Figure 2B for *ctt1* and Supplementary Figure S2C for *gpd1*). In cells lacking Top1, Pol II recruitment was more sustained than in wild-type cells (compare 60 min in both backgrounds in Figure 2B). Similar results were obtained measuring phosphorylation of Pol II CTD at Ser2 at ORFs and terminators of stress genes (Figure 2C and Supplementary Figure S2D).

**Figure 3.**
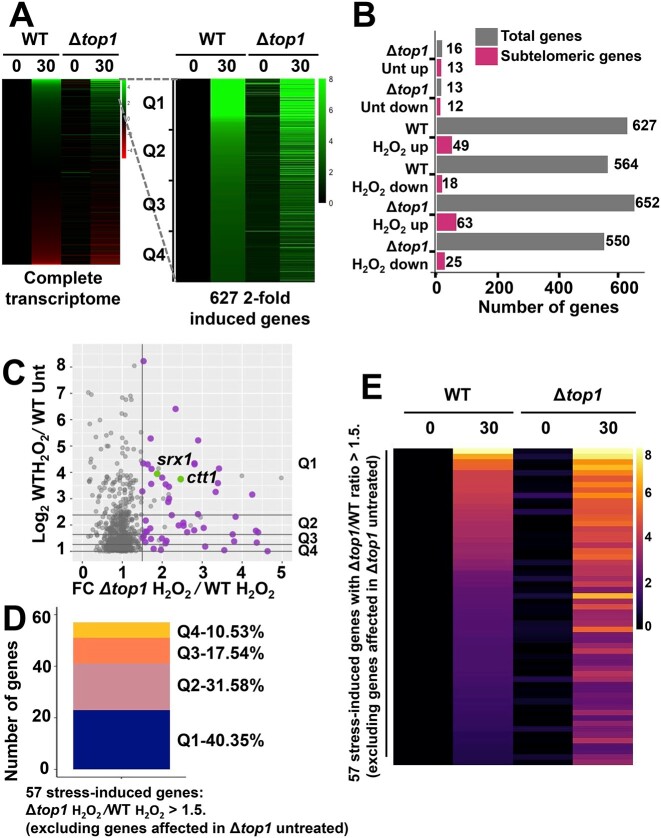
The general stress response is upregulated in Δ*top1* cells. (**A**) Highly induced stress response genes are upregulated in Δ*top1* cells. Total RNA from strains 972 (WT) and RF14 (Δ*top1*), treated or not with 1 mM H_2_O_2_ for 30 min, was sequenced using Illumina technology and analyzed as described under Materials and methods. Horizontal strips represent genes, and columns represent untreated or treated conditions of the indicated strains. The log_2_ changes in expression, relative to the untreated wild-type sample, are color-coded as shown in the bar (green for up-regulation and red for down-regulation). Genes were organized by their relative expression in the wild-type treated column. First panel shows the expression changes of the whole transcriptome, and second panel shows the 627 genes significantly up-regulated at least 2-fold in a wild type strain treated with 1 mM H_2_O_2_. Quartiles of expression are indicated, with Q1 being highest expressed genes in wild type treated sample. (**B**) Subtelomeric genes are over-represented among the up-regulated genes in Δ*top1* cells. Grey bars represent the number of genes significantly up- or down-regulated (2-fold) in each strain and treatment indicated. Pink bars indicate the number of affected genes (grey bars) that are consider subtelomeric. (**C**) Detail of a scattered plot showing the 627 genes indicated in A (right panel). Y axis shows expression of a wild-type treated relative to the wild-type untreated (expressed as log_2_). X axis shows the Δ*top1* to wild-type ratio of expression in treated samples. Purple and green dots highlight the significantly upregulated genes in a Δ*top1* relative to wild-type treated cells (total of 57 genes; upregulated 1.5-fold and *q*-value < 0.1, excluding genes upregulated in Δ*top1* in untreated conditions). The *srx1* and *ctt1* genes are indicated in green. (**D**) Among the 627 genes separated in 4 quartiles (Q1 to Q4) in A (right panel), 57 were upregulated in Δ*top1* relative to wild-type in treated cells (purple and green dots in C); the plot represents to which quartile of expression belongs each of the 57 upregulated genes. (**E**) Heat map showing the expression of the 57 upregulated genes in Δ*top1* relative to wild-type strain. Heatmap shows the log_2_ fold-change expression of the 57 selected genes (see C) in each condition relative to the wild-type untreated.

To further determine whether the whole stress program was enhanced in the absence of Top1, we analyzed the transcriptome of wild-type and Δ*top1* cells by RNA sequencing. We isolated RNA from cells cultures of wild-type and Δ*top1* strains grown in rich media, treated or not for 30 min with 1 mM H_2_O_2_; duplicates for all samples were processed. We obtained data from >5000 genes, covering most of the fission yeast transcriptome. More than 600 genes were up-regulated more than two fold in both strain backgrounds upon stress imposition, and close to 650 genes were down-regulated more than 2-fold (Figure 3A, left panel). In fission yeast, around 200 genes are located at the chromosomes' 100 kb end boundaries, which are called the subtelomeric areas (47). Under basal conditions, 16 and 13 genes were up- or down-regulated more than 2-fold in cells lacking Top1 relative to wild-type cells (Figure 3B), and almost all of them (13 and 12, respectively) were clustered at these subtelomeric domains (Figure 3B, pink bars). A similar effect has been reported in budding yeast upon deletion of *top1* (48). Inactivation of some silencing factors has also been shown to have a significant impact on these boundary chromosomal elements; that is the case of mutations in the HDAC Clr6 complex, which cause mis-regulation of genes at subtelomeres and Tf2 elements (49,50), and probably deletion of Top1 also causes defects in the chromatin architecture in these domains.

Regarding the response to H_2_O_2_, few more genes passed the threshold of 2-fold activation in Δ*top1* cells than in wild-type (652 versus 627, respectively). It is worth mentioning that many stress genes are located at subtelomeric regions, and they may be affected by Top1 deletion due to their proximity to the chromosomal ends. However, no significant changes in the number of subtelomeric genes in up- or down-regulated in Δ*top1* cells relative to wild-type cells were detected: a similar proportion of up-regulated genes, 49/627 and 63/652 in wild-type and Δ*top1* cells, are subtelomeric; the same occurs with down regulated genes (Figure 3B, pink bars).

An alternative hypothesis for the role of Top1 on transcription is that highly induced genes would benefit from the absence of the DNA topoisomerase. Top1 from *S. cerevisiae* has been localized to gene bodies of highly induced genes, probably associated to elongating Pol II (25). To test whether the absence of Top1 would affect more severely the expression of highly expressed genes, we divided the 627 genes up-regulated in a wild-type background upon stress in four quartiles, Q1 being highly induced upon stress, Q4 being only moderately induced (Figure 3A, right panel). In each quartile, we then measured the number of genes which were differentially up-regulated in cells lacking Top1 compared to wild-type cells by more than 1.5-fold; we subtracted those with altered values in untreated Δ*top1* cells to dismiss effects caused by proximity to the chromosomal ends. The remaining 57 genes were labelled in purple in Figure 3C. The *ctt1* and *srx1* genes, indicated in green in Figure 3C, which were accommodated in the upper quartile, Q1, fulfilled this criterion. As indicated in Figure 3D, most of the 57 genes differentially expressed in treated Δ*top1* cells compared to wild-type belonged to the upper Q1 quartile of highly expressed genes (40.3%); only few genes were present in the quartile of low-expressed genes, Q4 (10.5%). The heat map of the remaining 57 genes, listed in Supplementary Table S3, is shown as Figure 3E.

### The nucleosome architecture of highly induced stress genes remains open in Δ*top1* longer than in wild-type cells

**Figure 4.**
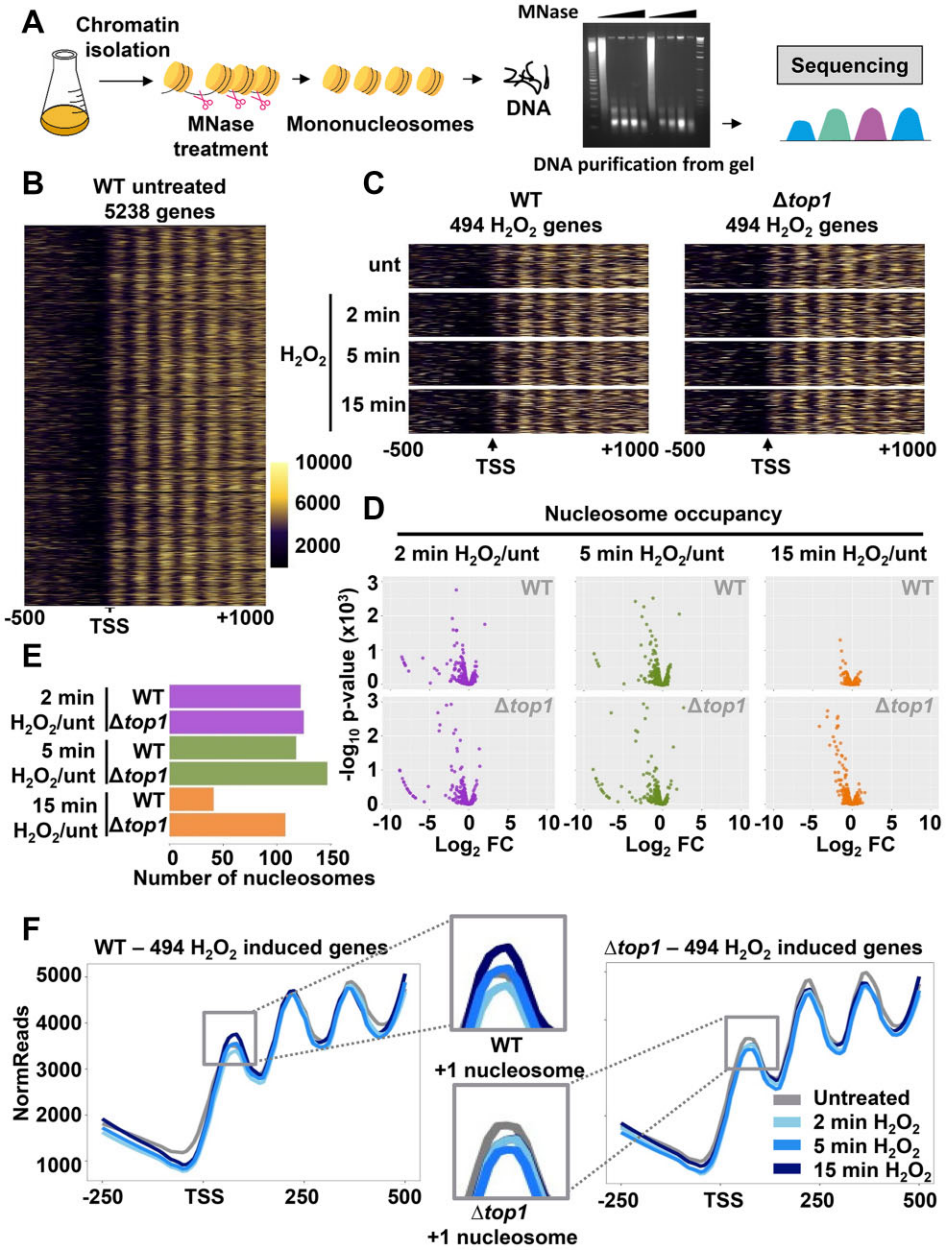
Nucleosome reassembly after stress imposition is slower in Δ*top1* cells. (**A**) Scheme depicting MNase-seq protocol. Mononucleosomes of strains 972 (WT) and RF14 (Δ*top1*) were isolated from cultures treated or not with 1 mM H_2_O_2_ for 2, 5 or 15 min. (**B**) Heatmap representing normalized reads from the position -50 to + 1000 base pairs relative to the TSSs (columns) of the total genome (rows). (**C**) Heatmaps showing normalized reads of the common 494 genes significantly induced in Δ*top1* and wild-type strains upon H_2_O_2_ treatment based on the transcriptomic analysis. Each heatmap represents the nucleosome occupancy in the indicated condition (untreated and 2, 5 and 15 min of H_2_O_2_ treatment) in wild-type (left panels) and Δ*top1* (right panels) strains. Rows represent individual genes and columns are the base pair distance to the TSSs (from −500 to +1000 bp). (**D**) Volcano plots depicting nucleosome occupancy changes of the 494 genes commonly induced upon peroxide treatment. Each dot represents a nucleosome identified by DANPOS2. Only nucleosomes in the −50 to +500 relative to the TSS of the 494 genes are analyzed. X axis of each plot shows the log_2_ fold change (FC) of nucleosome occupancy in the indicated time points after peroxide treatment compared with the untreated. Upper three panels: wild-type strain; bottom three panels: Δ*top1* strain. Y axis represent the log_10_*P*-value. (**E**) Number of nucleosomes from the 494 genes, as shown in D, that present a decrease of ≤1.5 fold change in nucleosome occupancy and are statistically significant. (**F**) Line plots depicting average reads of the 494 stress induced genes in wild-type (left panel) and Δ*top1* (right panel) strains. Lines correspond to each one of the conditions analyzed: untreated and 2, 5 and 15 min after H_2_O_2_ treatment.

To gain a whole-genome view of the chromatin architecture at stress genes, we purified MNase-derived mononucleosomes of biological duplicates of wild-type and Δ*top1* rich media cultures, under untreated conditions or 2, 5 or 15 min after 1 mM H_2_O_2_, and sequenced them (Figure 4A). We first monitored the similarity between replicates of each experimental condition by calculating the Pearson coefficients, which were higher than 0.93 for all couples (Supplementary Figure S3). We then averaged the nucleosome profile of all *S. pombe* genes, aligning nucleosomal arrays based on the published TSSs. Figure 4B shows the profile of nucleosome occupancy of wild-type untreated cells, from −500 to +1000 base pairs relative to the TSSs (indicated with an arrow). As previously shown (51), clear NDRs were prominent in many *S. pombe* genes, with the nucleosome arrays downstream of the TSSs emanating from the NDRs. No apparent differences in these nucleosome patterns were observed among the 8 different samples (2 strains and 4 biological conditions) when evaluating the 5000 genes (unpublished data). When the ∼500 genes upregulated upon H_2_O_2_ in both wild-type and Δ*top1* strains based on our transcriptomic analyses, which are listed in Supplementary Table S4, were analyzed, the nucleosome pattern was also apparently similar (Figure 4C).

We then used the DANPOS2 algorithm (43) to identify the position and assign a quantitative value to each nucleosome of these 494 genes. We chose to compare the values of ∼1500 nucleosomes downstream the TSS of these differentially expressed genes. As shown in Figure 4D with Volcano plots, in which each dot represents a nucleosome located around the TSS (−50 to +500 bp relative to the TSS), dozens of nucleosomes were more weakly positioned 2 and 5 min after H_2_O_2_ stress relative to untreated conditions, both in a wild-type and Δ*top1* strain. 15 min after stress imposition, only few nucleosomes were still partially evicted in a wild-type background, whereas the number of nucleosomes which remained significantly less occupied relative to untreated conditions was much higher in cells lacking Top1 (Figure 4D and E; Supplementary Table S5).

We averaged nucleosome occupancy nearby the TSS of these ∼500 H_2_O_2_-upregulated genes. As shown in Figure 4F, the NDR and three nucleosomes were clearly positioned and visualized when analyzing the area between −250 to +500, TSS being +1, under untreated conditions (grey line). In a wild-type background (left panel), 2 and 5 min after H_2_O_2_ treatment de number of reads corresponding to the first nucleosomes of these ∼500 stress genes decreased relative to the untreated condition in wild-type cells, and the reads returned to the starting levels 15 min after stress imposition; this was particularly apparent in the + 1 nucleosome (dark blue line in inset of WT + 1 nucleosome in Figure 4F). This would suggest that nucleosomes were partially evicted from the gene bodies of stress genes, returning to the closed conformation at longer times. Similar observations were obtained for the reads/nucleosomes of Δ*top1* at short times. Nevertheless, nucleosomes did not return to the starting position 15 min after stress, indicating that the chromatin remained open at longer times. The delay in closing chromatin of cells lacking Top1 was particularly apparent in the +1 nucleosome of the stress genes (dark blue line in inset of Δ*top1* + 1 nucleosome in Figure 4F). Of note, the differences observed for the averaged nucleosome maps of the 494 stress genes between wild-type and Δ*top1* strains was more dramatic for the highly expressed genes of the quartil 1, than for the genes of the other quartils (unpublished data).

**Figure 5.**
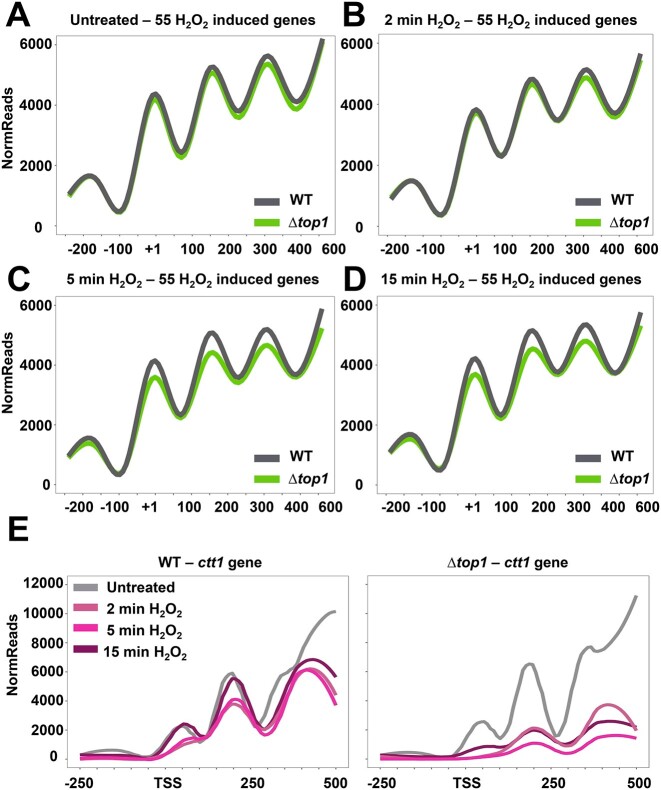
Composite plots of relative nucleosome occupancy of 972 (WT) and RF14 (Δ*top1*) strains. Line plots comparing average nucleosome occupancy of wild-type and Δ*top1* under the different conditions: untreated (**A**), and after 2 (**B**), 5 (**C**) and 15 min (**D**) of H_2_O_2_ treatment. Each line is the average normalized reads of the stress induced genes described in Figure 3E. Genes were aligned to the +1 nucleosome (first after the TSS). (**E**) Nucleosome occupancy of catalase (*ctt1*) gene in wild-type (left panel) and Δ*top1* (right panel) in untreated and H_2_O_2_ treatment (2, 5 and 15 min).

We then analyzed the nucleosome maps of the 55 genes differentially up-regulated in cells lacking Top1 compared to wild-type cells, based on our transcriptomic analysis (listed in Supplementary Table S6); only 2 of the genes of Figure 3E were discarded due to the small number of reads mapping to their locations in our nucleosome-sequencing analysis. Since the TSS coordinates of some of these genes seemed to be improperly annotated, we used the + 1 nucleosome center to synchronize all their nucleosome maps (+1 in Figure 5A). The average nucleosome position became fuzzy after stress imposition in both strain backgrounds (Supplementary Figure S4A), but this open configuration seemed to be fully maintained in cells lacking Top1 (Figure 5AD). Thus, when we averaged the +1 nucleosome of these 55 stress genes, it became very clear that the nucleosome maps of wild-type and Δ*top1* cells were undistinguishable under untreated conditions and 2 min after stress imposition (Figure 5A, B), but they differed at longer times (Figure 5C, D), indicating that the +1 to +3 nucleosomes had returned to their original position in wild-type cells, but remained fuzzy in strain Δ*top1*. Importantly, the average nucleosome map of 55 control genes (listed in Supplementary Table S7) was unaffected by stress in both wild-type or Δ*top1* cells (Supplementary Figure S4B). From these experiments, we conclude that the absence of Top1 impairs nucleosome reassembly after the transcriptional machinery has passed along the stress genes, and consequently these genes remain open longer than in wild-type cells.

### Nucleosome architecture around the *ctt1* gene

Since the *ctt1* gene was clearly up-regulated to a larger extent in a Δ*top1* background than in wild-type cells in our transcriptomic analysis (2.46 Δ*top1-*to-WT induction ratio), we used the nucleosome sequencing data to test whether the chromatin architecture at *ctt1* before and after stress is different in both strains. As previously reported using nucleosome scanning (amplification with pairs of overlapping primers of purified nucleosomes and analysis by quantitative PCR) (15), the nucleosome landscape of the *ctt1* gene was altered upon stress imposition, probably due to partial nucleosome eviction caused by the elongating transcriptional machinery (Figure 5E, left panel). Thus, the well-established nucleosomes downstream of the TSS (+1 to +3 shown in Figure 5E) were weakly positioned 2 and 5 min after stress imposition. Nucleosomes + 1 and + 2 fully returned to the starting position 15 min after H_2_O_2_ stress in a wild-type background. The +1 to +3 *ctt1* nucleosomes of cells lacking Top1 also displayed a very stable position prior to stress, which was largely lost 2 and 5 min after H_2_O_2_ stress; importantly, 15 min after H_2_O_2_ the nucleosomes were far from being reassembled to their original localization (Figure 5E, right panel). These experiments suggest that the chromatin at the *ctt1* gene remains open long after stress imposition in cells lacking Top1. A very similar pattern was observed at other stress genes such as *srx1, trr1, tpx1* or *caf5* (Supplementary Figure S5A). For each gene, the number of nucleosome reads in the 0–500 bp area relative to the TSS after 15 min of peroxide stress was lower in cells lacking Top1 that in the wild-type strain (Supplementary Figure S5B), suggestive of an open chromatin configuration.

As shown above, the nucleosome landscape of the *ctt1* gene remained open after 15 min of stress imposition in cells lacking Top1. To confirm that nucleosomes eventually returned to their positions in this background, we isolated mononucleosomes from wild-type and Δ*top1* cultures 1 and 2 h after H_2_O_2_ stress, and compared them with those of shorter time points by nucleosome scanning. Briefly, DNA from the mononucleosome samples was PCR-amplified with overlapping pairs of primers covering ∼0.8 kb of the *ctt1* gene, as described before (15). As shown in Supplementary Figure S6, the +1 to +3 nucleosomes downstream of the TSS were largely repositioned in Δ*top1* cells 2 h after stress imposition.

### Genetic interaction between *top1* and the chromatin remodeler-coding genes *hrp1, hrp3* and *snf22*

Stress-dependent nucleosome architecture is therefore affected by deletion of *top1*. A similar effect could be expected from defects in chromatin remodelers. In fact, it was suggested that chromatin remodelers use ATP hydrolysis to drive local changes in the DNA twist, and therefore have an impact on DNA topology, at least *in vitro* (52). We tested the effect on oxidative stress tolerance and transcription of three remodelers, previously connected to other Atf1 functions: Snf22, Hrp1 and Hrp3.

**Figure 6.**
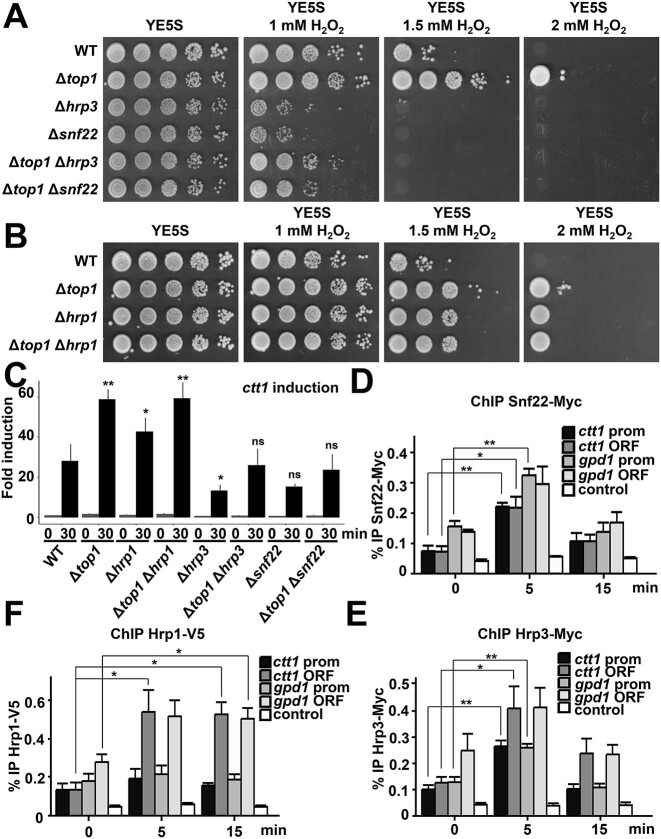
Cells lacking the nucleosome remodeler Hrp1 are resistant to oxidative stress. (**A**) Cells lacking nucleosome remodelers Hrp3 or Snf22 are sensitive to H_2_O_2_. Experiments were performed as in Figure 1A with strains 972 (WT), RF14 (Δ*top1*), IV83 (Δ*hrp3*), IV84 (Δ*snf22)*, RF117 (Δ*top1* Δ*hrp3*) and RF118 (Δ*top1* Δ*snf22*). (**B**) Cells lacking nucleosome remodeler Hrp1 are resistant to H_2_O_2_. Experiments were performed as in Figure 1A, using strains 972 (WT), RF14 (Δ*top1*), IV69 (Δ*hrp1*) and RF99 (Δ*top1* Δ*hrp1*). (**C**) Expression of *ctt1* was analyzed by quantitative PCR in the strains from A and B. Amplification with *act1* primers was used as a control for normalization. Bar plots represent the mean value and SD from biological triplicates. Significant differences between each mutant strain and the wild-type strain after peroxide treatment were determined by a two-sided *t*-test (*P< 0.05, **P< 0.01). (**D–F**) Snf22, Hrp3 and Hrp1 recruitment to the promoters and ORFs of stress genes upon stress imposition. ChIP experiments as in Figure 1D using anti-Myc or anti-V5 antibodies were performed from cultures of strains JA2653 (*snf22-myc*) (**D**), JA2777 (*hrp3-myc*) (**E**) or RB175 (*hrp1-V5*) (**F**), using primers covering promoter or ORF regions of *ctt1* and *gpd1*. Primers of a *mtDNA* region were used as control. Bar plots represent the mean value and SD from biological triplicates. Significant differences were determined by a two-sided *t*-test (*P< 0.05, **P< 0.01).

Regarding tolerance to oxidative stress, cells lacking Hrp3 or Snf22 are severely sensitive to oxidative stress (Figure 6A), and the combination of gene deletions of Δ*top1* with either Δ*hrp3* or Δ*snf22* yielded strains with intermediate phenotypes, suggesting that Top1 and these chromatin remodelers affect oxidative stress tolerance through independent mechanisms.

On the contrary, deletion of *hrp1* yielded a strain resistant to H_2_O_2_, almost to the same extent as Δ*top1* (Figure 6B). The resistance phenotypes of the single deletes did not add in the double deletion strain, suggesting that Top1 and Hrp1 contribute to H_2_O_2_ tolerance through a similar mechanism.

H_2_O_2_ tolerance of the strains lacking Hrp1, Hrp3 or Snf22 perfectly correlated with the expression levels of the *ctt1* and *srx1* genes, as shown in Figure 6C for *ctt1* and Supplementary Figure S7A for *srx1*. Thus, the H_2_O_2_–dependent expression of the stress genes *ctt1* and *srx1* is lower than in wild-type cells in the case of Δ*hrp3* or Δ*snf22*, and is significantly higher in the case of strain Δ*hrp1*.

To test by ChIP the recruitment at promoters and ORFs of stress genes of these chromatin remodelers, we modified their endogenous genes and checked that the tagged proteins remained functional and maintained wild-type tolerance to H_2_O_2_ (Supplementary Figure S7B). While Top1-HA was present at the ORFs of stress genes 5 and 15 min of H_2_O_2_ stress (Figure 2A), Snf22-Myc and Hrp3-Myc were transiently recruited at both promoters and ORFs of the *ctt1* and *gpd1* genes 5 min after stress imposition (Figure 6D, E). Hrp1-V5 was also recruited to DNA, mainly at ORFs but also at promoters, but remained at stress genes 15 min after stress (Figure 6F), similar to Top1-HA. The recruitment of Hrp1 and Top1 to stress genes was not significantly impaired in the absence of each other (Supplementary Figure S7C, D). In combination, these results suggest that Snf22 and Hrp3 have earlier and transient roles at stress genes, such as opening the chromatin to promote transcription progression, whereas Top1 and Hrp1 may be recruited to favor a later step in transcription, such as chromatin closing.

**Figure 7.**
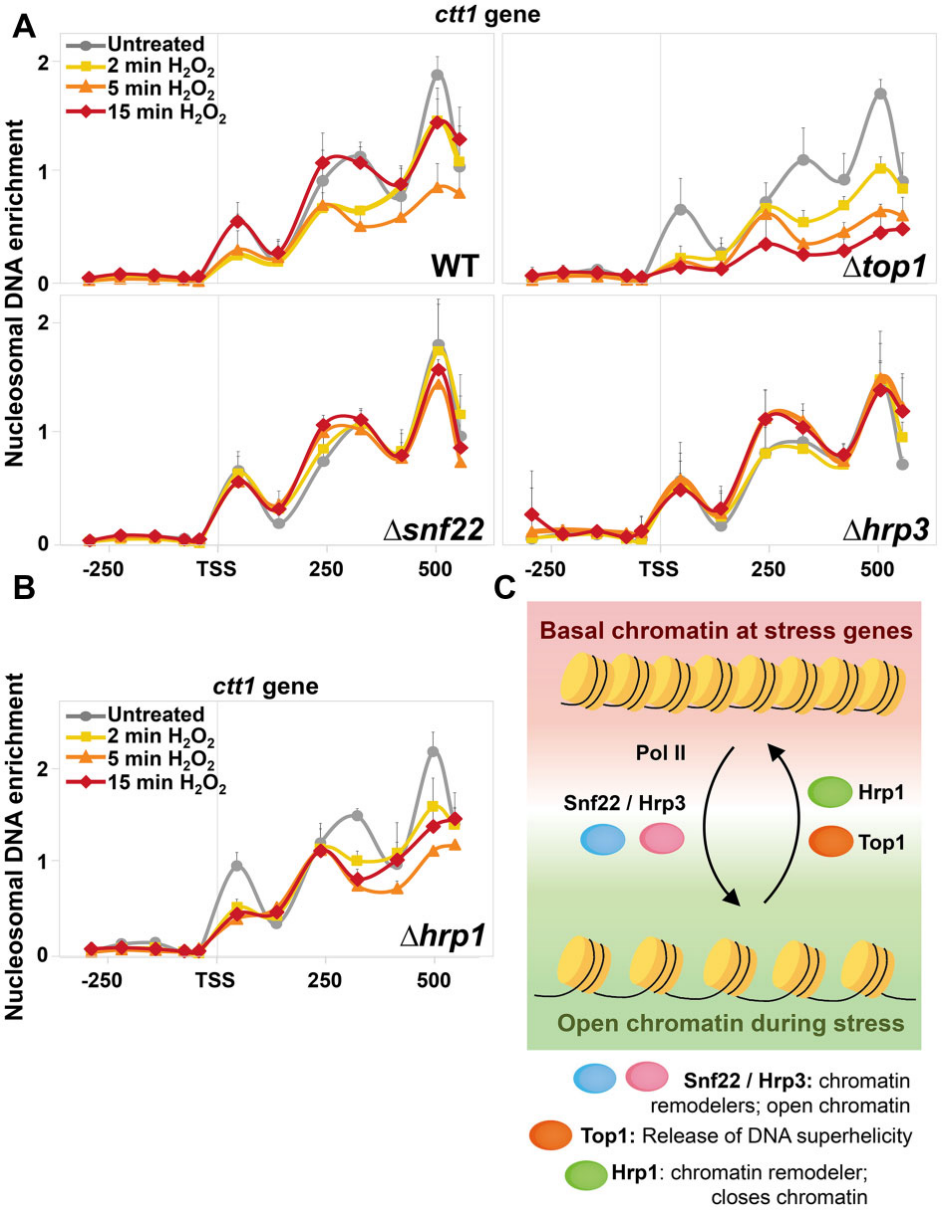
Cells lacking the nucleosome remodelers Snf22, Hrp3 or Hrp1 display altered nucleosome landscapes after stress imposition relative to wild-type cells. (**A, B**) Nucleosome scanning of the *ctt1* gene before (in grey; circles) and after (2 min, in yellow, squares; 5 min, in orance, triangles; 15 min, in red, diamonds) 1 mM H_2_O_2_ stress imposition was performed in strains 972 (WT), RF14 (Δ*top1*), IV84 (Δ*snf22*), IV83 (Δ*hrp3*) and IV69 (Δ*hrp1*). Lines represent average of biological triplicates with error bars (SD). (**C**) Scheme depicting the proposed role of the Top1 topoisomerase and the chromatin remodelers Snf22, Hrp3 and Hrp1 in nucleosome architecture at stress genes. See text for details.

To confirm this hypothesis, we isolated mononucleosomes of cells lacking these chromatin remodelers before and after H_2_O_2_ stress, and compared them with those of wild-type and Δ*top1* cells by nucleosome scanning. As shown in Figure 7A, cells lacking Snf22 and Hrp3 were largely unable to open the chromatin structure at the *ctt1* gene upon stress imposition. On the contrary, in cells lacking Hrp1 the +1 to +3 nucleosomes downstream of the TSS were largely evicted 2 and 5 min after H_2_O_2_ treatment, and this nucleosome architecture remained open after 15 min (Figure 7B), while nucleosomes were largely repositioned in wild-type cells at this time point (Figure 7A, top left panel). Of note, the nucleosomes of Δ*hrp1* cells were also repositioned at longer time points (60 and 120 min; Supplementary Figure S7E), slightly earlier that in cells lacking Top1 (Supplementary Figure S6). We used ChIP to analyze whether the Δ*hrp1* strain displayed a more sustained recruitment of the transcriptional machinery, using antibodies against Pol II Ser2-P. As shown in Supplementary Figure S7F, the stress-dependent recruitment of Ser2-phosphorylated Pol II to the *ctt1* gene was significantly higher in cells lacking Hrp1 than in wild-type cells.

## Discussion

In biology, all signal-dependent processes are based on activating activities but also on inactivating ones, to return to the original state. Environmental stresses strongly rely on transcriptional-driven expression programs for survival. We show here that the lack of Top1 impairs nucleosome reassembly during stress recovery, and Pol II elongation is more sustained. In the absence of Top1, it is likely that transcription-dependent DNA supercoiling is higher than in wild-type cells; our experiments demonstrate that nucleosome reassembly is slower in this context, so that Pol II displays a more prolonged presence at stress genes in Δ*top1* cells.

Top1 in fission yeast has been proposed by ChIP sequencing to accumulate at the boundaries of gene bodies, with a similar profile to the essential Top2 (37). Thus, it was proposed that Top1 activity participates in efficient nucleosome disassembly at gene promoters. A recent report in budding yeast demonstrated by ChIP-on-chip that Top2 accumulates upstream and downstream of the gene bodies, and Top1 is confined within the ORFs, accumulating with a profile very similar to Pol II (25). In the same line, we shown here by ChIP that the presence of Top1 at ORFs (but not promoters) of stress genes is augmented upon stress imposition (Figure 2A).

Our analysis seems to indicate that the absence of Top1 probably affects the basal and induced expression of genes located at subtelomeres, many of which are stress genes. But we have also shown that highly induced genes are more prone to be affected by the lack of Top1 (Figure 3D). In line with this view, the relative chromosomal locations of three classical stress genes, *ctt1, srx1* and *hsp9*, which are over-induced in cells lacking Top1, are shown in Supplementary Figure S8; only *ctt1* is located close to the left end of chromosome III. Of note, the absence of Top1 significantly affects nucleosome re-positioning after stress at the *ctt1* gene (Figure 5E); its proximity to the chromosomal end could contribute to this dramatic effect.

We show here that the endonuclease activity of Top1 is required for its function, since cells expressing a catalytically dead mutant displayed the same phenotypes as Δ*top1* cells. The experiments shown here make us hypothesize that removal of supercoils at gene bodies of stress genes by Top1 may stimulate nucleosome re-assembly by the chromatin remodeler Hrp1 to return to the starting and closed nucleosomal architecture. We show that Hrp3 and Snf22 are recruited early to promoters and ORFs of stress genes but are not present at later time points, and in their absence stress dependent chromatin remodeling is largely impaired (Figure 7C). We propose a model by which Hrp3 and Snf22 collaborate with the transcriptional machinery during the first rounds of transcription after stress imposition to promote nucleosome eviction, sliding or fuzziness, as suggested in Figure 7C. On the contrary, Hrp1 and Top1 are mainly recruited at ORFs of stress genes, where they remain at later time points; in their absence, the chromatin stays open longer than in wild-type cells (Figure 7C). Interestingly, a connection between DNA topoisomerases and Hrp1 in nucleosome dynamics at promoters was proposed by the group of Ekwall (37). Furthermore, and in line with our work, opposite roles of Hrp1 and Snf22 or Hrp3 have been proposed in triggering homologous recombination at *ade6-M26*, with Hrp1 playing a role in suppressing chromatin opening and Snf22/Hrp3 aiding to establish an open chromatin configuration (21). Also related to our proposal, a role for fission yeast's topoisomerases in nucleosome positioning at the *fbp1* gene has also been demonstrated (53). Based on our work and on previous literature, we propose that DNA topology and chromatin remodelers collaborate to establish the basal and stress-induced chromatin landscapes at gene bodies. In the absence of Top1, we also propose that DNA helical tension at stress genes may be enhanced but nucleosome reassembly impaired, and this second feature prevails and causes more sustained Pol II -dependent transcription than in wild-type cells.

## Supplementary Material

gkad1066_Supplemental_Files

## Data Availability

The data underlying this article are available in the article and in its online supplementary material. Original data such as images and blots have been deposited in Figshare (https://doi.org/10.6084/m9.figshare.22293640). Regarding RNA-seq and MNase/nucleosome sequencing, the data underlying this article are available in GEO (RNA-seq: GSE227183; MNase-sep: GSE227182).
