# Supplementary material for: Topoisomerase 1 facilitates nucleosome reassembly at stress genes during recovery
Source: Nucleic Acids Res. 2023 Nov 13;51(22):12161–73. doi: 10.1093/nar/gkad1066 (PMC10711424; doi:10.1093/nar/gkad1066)
Supplement: gkad1066_Supplemental_Files [file gkad1066_supplemental_files.zip › vega_supp_material_ver2.docx]

**SUPPLEMENTAL INFORMATION**

**Topoisomerase 1 facilitates nucleosome reassembly at stress genes during recovery**

Montserrat Vega, Rubén Barrios, Rodrigo Fraile, Kevin de Castro Cogle, David Castillo, Roger Anglada, Ferran Casals, José Ayté, Ernesto Lowy-Gallego and Elena Hidalgo

It includes:

Figures S1 to S8

Tables S1 and S2

References

**
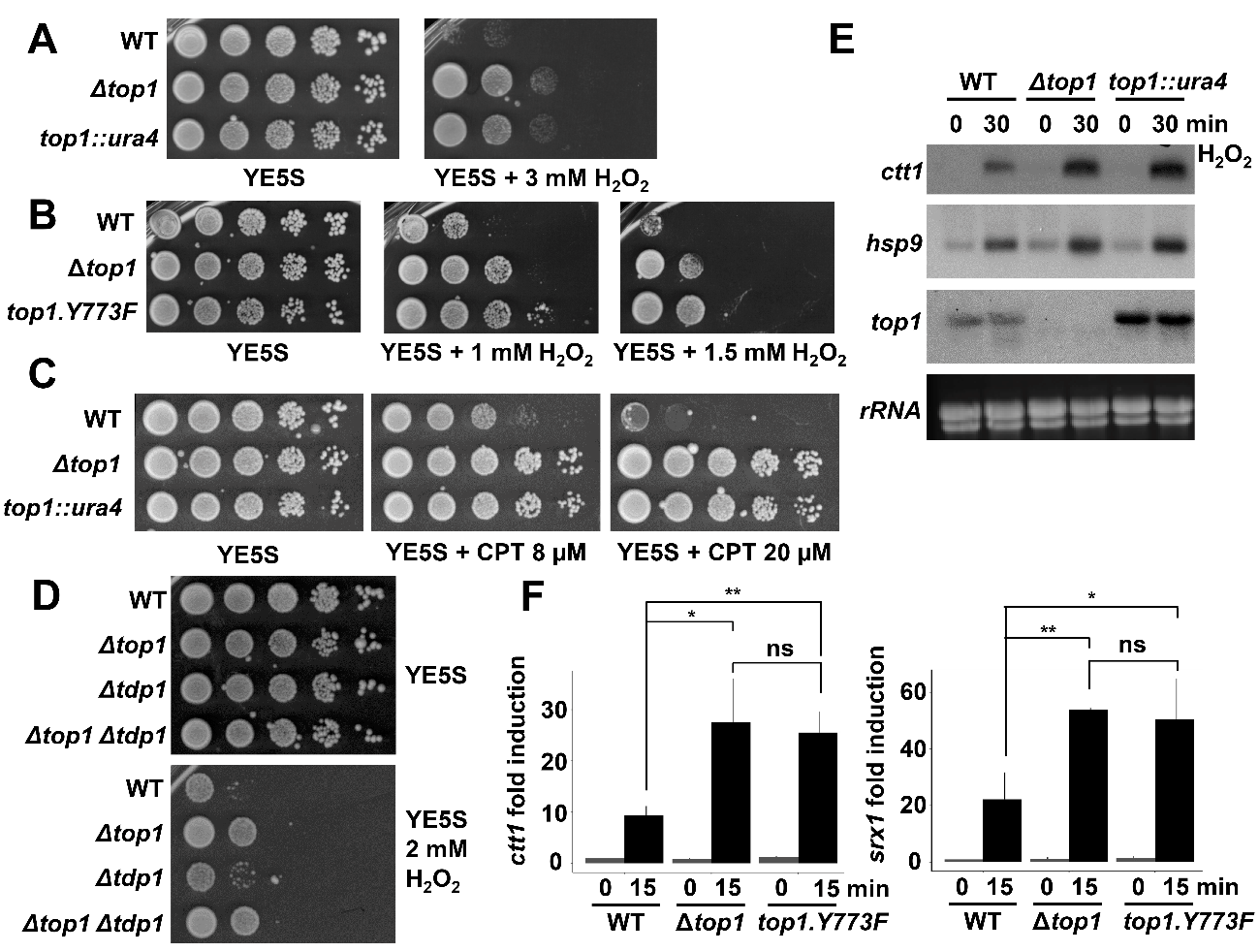
**

**Supplementary Figure S1**. Cells expressing a truncated version of Top1 lacking the catalytic domain are also resistant to oxidative stress. (**A**) Experiments were performed as in Figure 1A with the strains 972 (WT), RF14 (Δ*top1*) and RF56 (Δ*top1::ura4*). (**B**) Experiment performed as in Figure 1A, using strains 972 (WT), RF14 (Δ*top1*) and MV118 (*top1.Y773F*). (**C**) Experiment performed as in Figure 1A, using plates with the indicated concentrations of camptothecin (CPT). (**D**) Experiments performed as in Figure 1A, using strains 972 (WT), RF14 (Δ*top1*), RF120 (Δ*tdp1*) and RF119 (Δ*top1* Δ*tdp1*). (**E**) Total RNA from cultures of the strains 972 (WT), RF14 (Δ*top1*) and RF56 (*top1::ura4*), treated or not with 1 mM H_2_O_2_, was obtained and analyzed by Northern blot with labeled *ctt1*, *hsp9* and *top1* probes. rRNA was used as loading control. (**F**) Expression of *ctt1* and *srx1* was analyzed by quantitative PCR in strains as in **A**. Amplification with *act1* primers was used as a control for normalization. Each bar represents the mean value and SD from biological triplicates. Significant differences between samples were determined by a two-sided t-test (*p<0.05, **p<0.01, ns, non-significant).

**
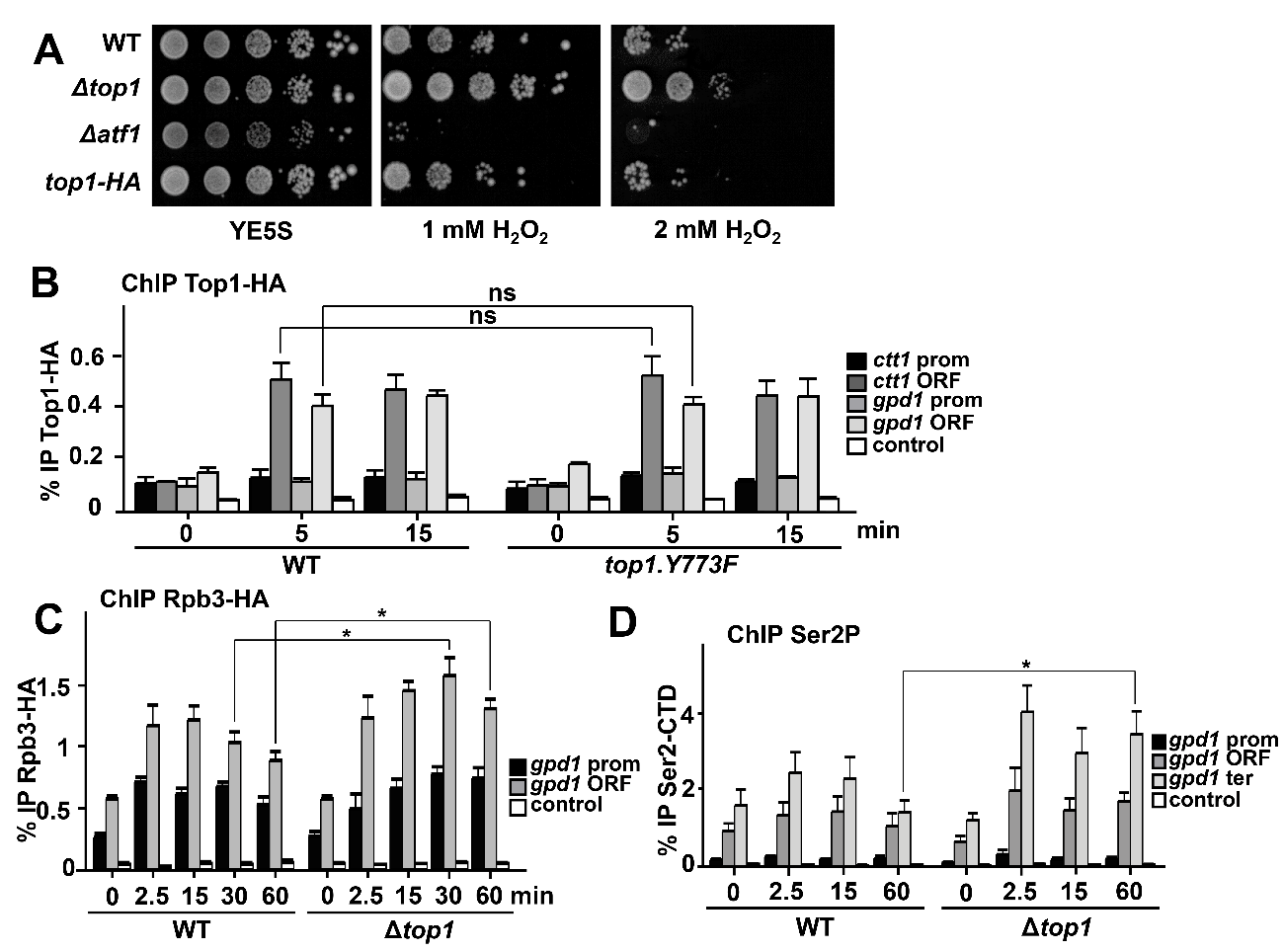
**

**Supplementary Figure S2**. Recruitment of Top1.Y773F-HA and Pol II to stress genes. (**A**) Experiments were performed as in Figure 1A using the strains 972 (WT), RF14 (Δ*top1*), MS98 (Δ*atf1*) and RF92 (*top1-HA*). (**B**) Catalytically-dead version of Top1 is similarly recruited to stress genes after oxidative stress. ChIP experiments as in Figure 1D using anti-HA antibodies were performed from cultures of strains RF92 (WT *top1-HA*) and RB174 (*top1*.*Y773F-HA*), using primers covering promoter or ORF regions of *ctt1* and *gpd1*. Primers of a *mtDNA* region were used as control. Each column represents the mean value and SD, calculated from three biological replicates. Significant differences were determined by a two-sided t-test (ns, non-significant). (**C**) Recruitment of Pol II to the *gpd1* gene. ChIP experiments as in Figure 1D using anti-HA antibodies were performed from cultures of strains JF5 (Rpb3-HA) and RF96 (∆*top1* Rpb3-HA), using primers covering promoter or ORF regions of *gpd1*. Primers of a *mtDNA* region were used as control. Each bar represents the mean value and SD from three biological replicates. Significant differences were determined by a two-sided t-test (*p<0.05). (**D**). Recruitment of elongating Pol II to the *gpd1* gene. ChIP experiments as in Figure 1D using anti-Ser2-P antibodies were performed from cultures of strains 972 (WT) and RF14 (Δ*top1*), using primers covering promoter, ORF and terminator regions of *gpd1*. Primers of a *mtDNA* region were used as control. Each bar represents the mean value and SD from biological triplicates. Significant differences were determined by a two-sided t-test (*p<0.05).

**
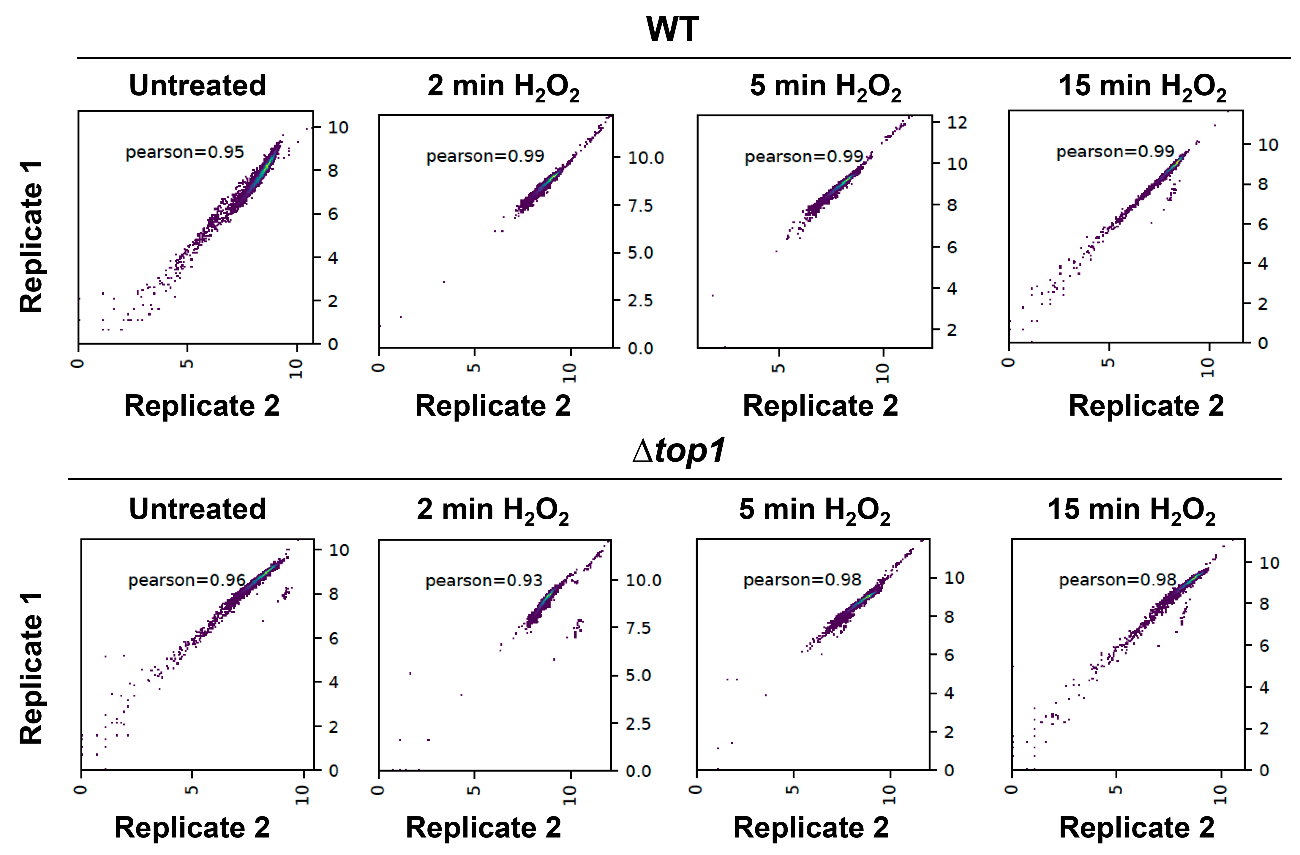
**

**Supplementary Figure S3**. Pairwise scatter plots with correlation coefficients for MNase-seq replicates. Pairwise scatter plots based on genome read coverages (in bins of 1000 bps). Each dot represents the coverage, in natural log scale, for each one of the bins. Axis represents the coverage in each of the replicates. Plots include the Pearson correlation coefficients for each comparison. Read coverages as well correlations were calculated using the bioinformatics deepTools suite.

**
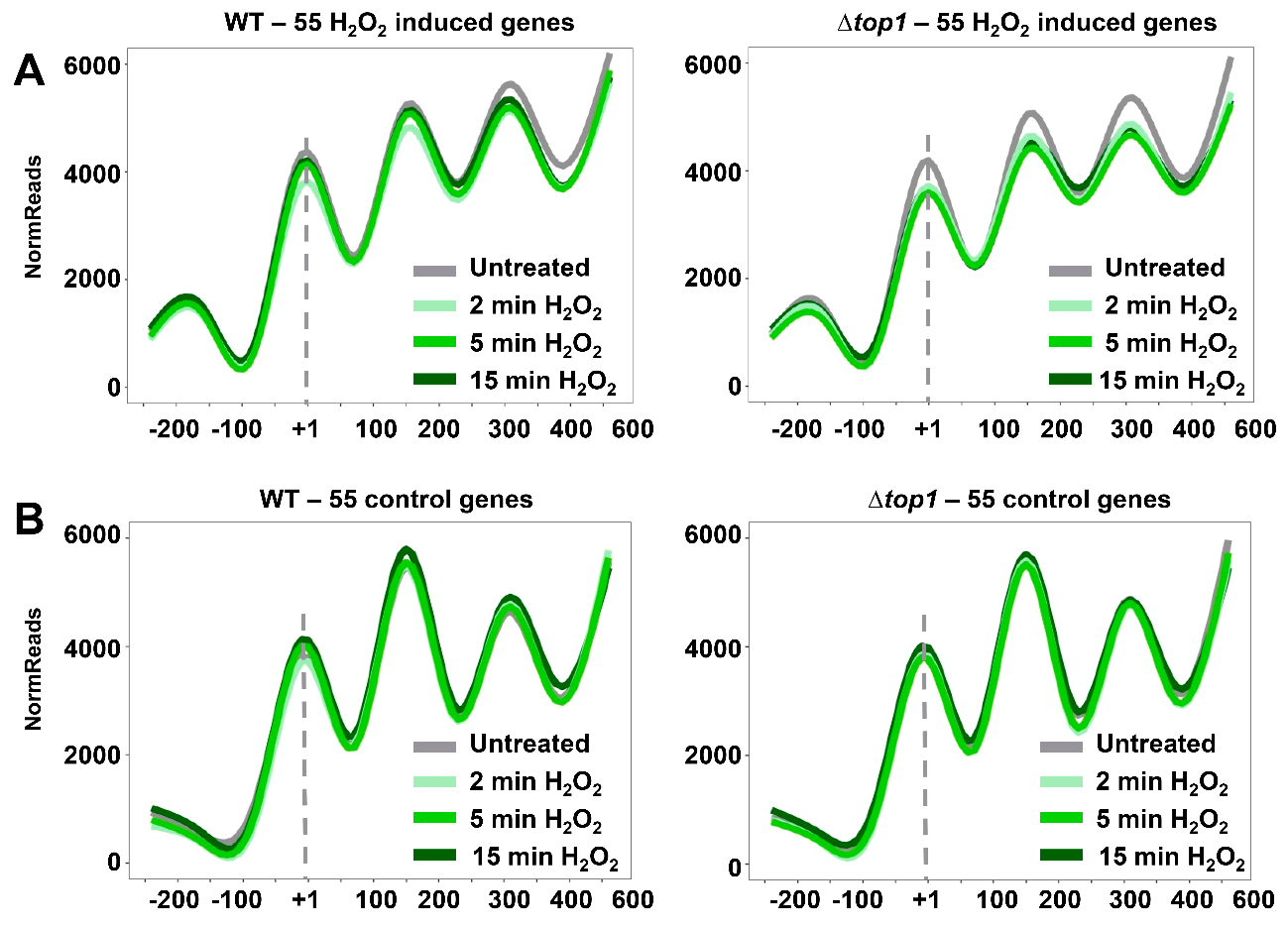
**

**Supplementary Figure S4.**  Nucleosome occupancy of highly expressed and unaffected H_2_O_2_ genes. (**A**) Line plots comparing nucleosome occupancy in a wild-type (left panel) and Δ*top1* (right panel). Each line represents the average of normalized reads of 55 stress induced genes, all of them included in the 55 genes of Figure 3E, in untreated conditions and after 2, 5 and 15 minutes of H_2_O_2_ treatment. Nucleosomes were aligned at their +1 nucleosome (the first one after the TSS). (B) Nucleosome occupancy of 55 control genes. Line plots comparing the nucleosome position of 55 control genes in a wild-type (left panel) and Δ*top1* (right panel) strains. Control genes did not present expression changes upon H_2_O_2_ treatment based on the transcriptomic analysis (Figure 1A). Nucleosomes were aligned at their +1 nucleosome (the first one after TSS).

**
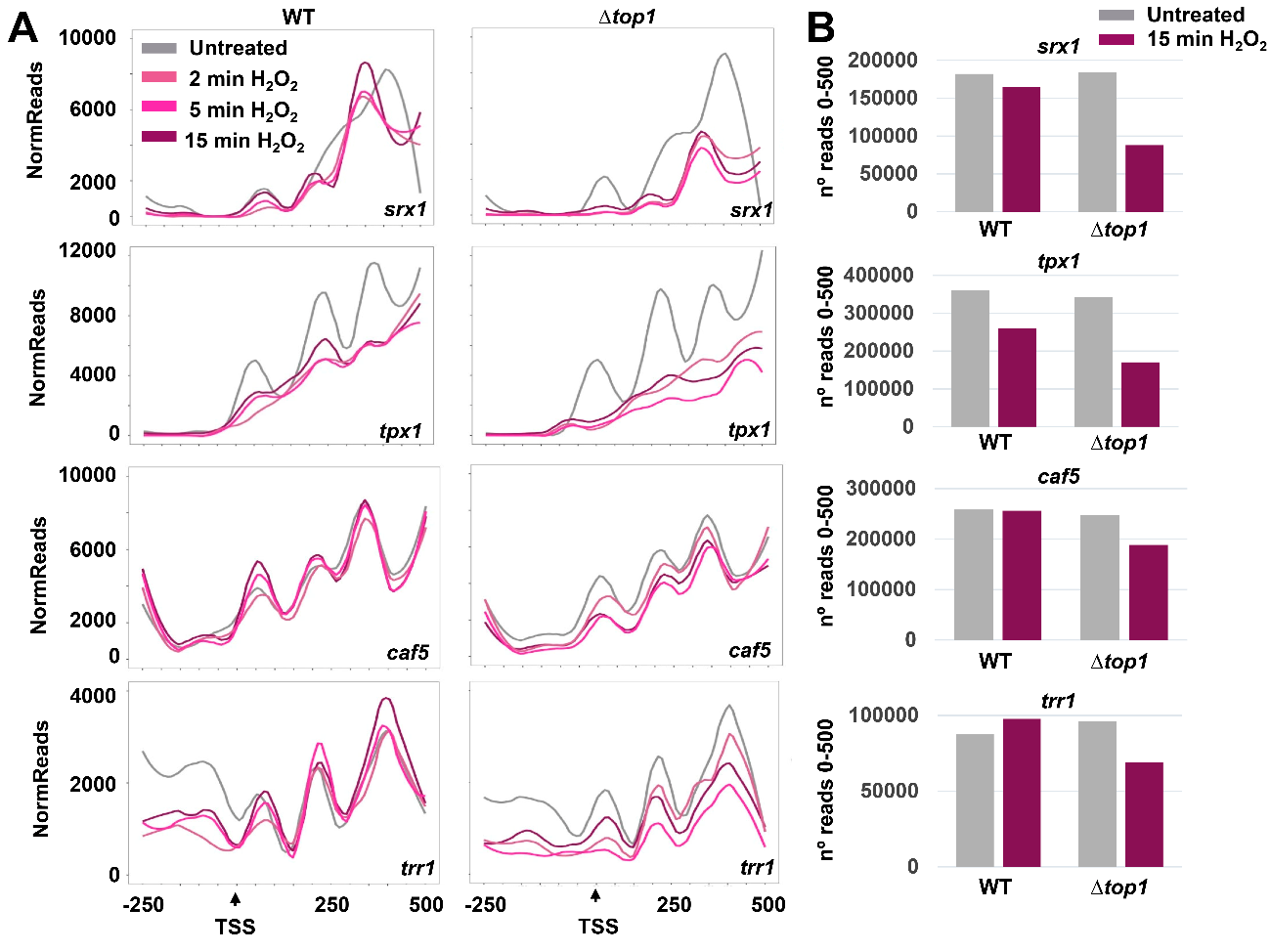
**

**Supplementary Figure S5.** Nucleosome occupancy of individual representative stress genes. (**A**) Line plots represent normalized reads of *srx1*, *tpx1*, *caf5* and *trr1* genes from -250 to +500 bp relative to the TSS in a wild-type strain (left panels) and Δ*top1* (right panels) strains. (**B**) Barplots comparing the total number of reads from TSS to +500 pb in wild-type and Δ*top1* in untreated (grey bar) *versus* 15 minutes after H_2_O_2_ treatment (magenta).


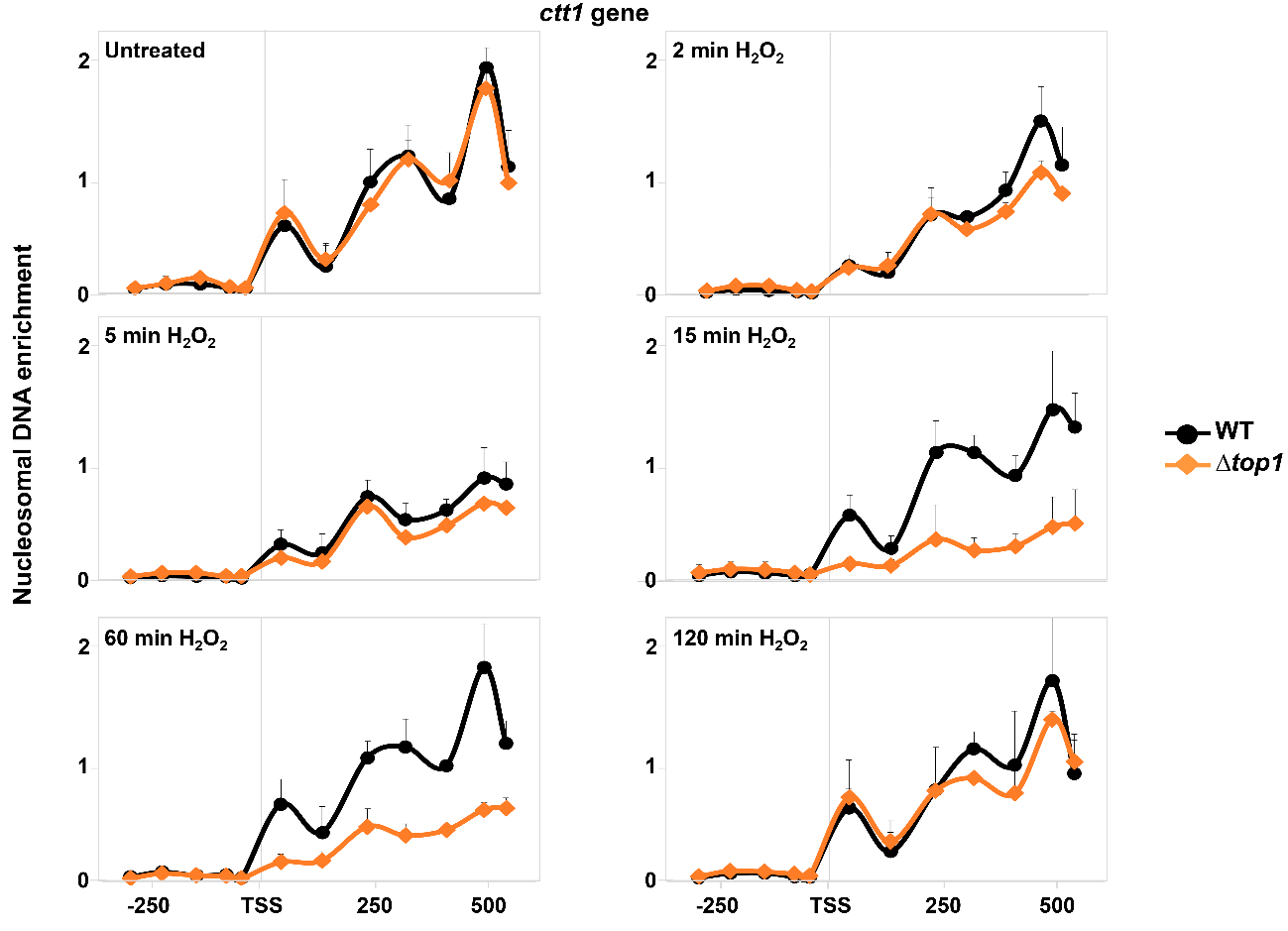


**Supplementary Figure S6.** Nucleosome scanning analysis of *ctt1* gene of wild-type vs. Δ*top1*, long times. Nucleosome scanning performed as in Figure 7A-B with the strains 972 (WT) and RF14 (Δ*top1*) before and after peroxide treatment (2, 5, 15, 60 and 120 minutes). Lines represent average of biological triplicates with error bars (SD) for wild-type (in black, circles) and Δ*top1* (in orange, diamonds) strains.


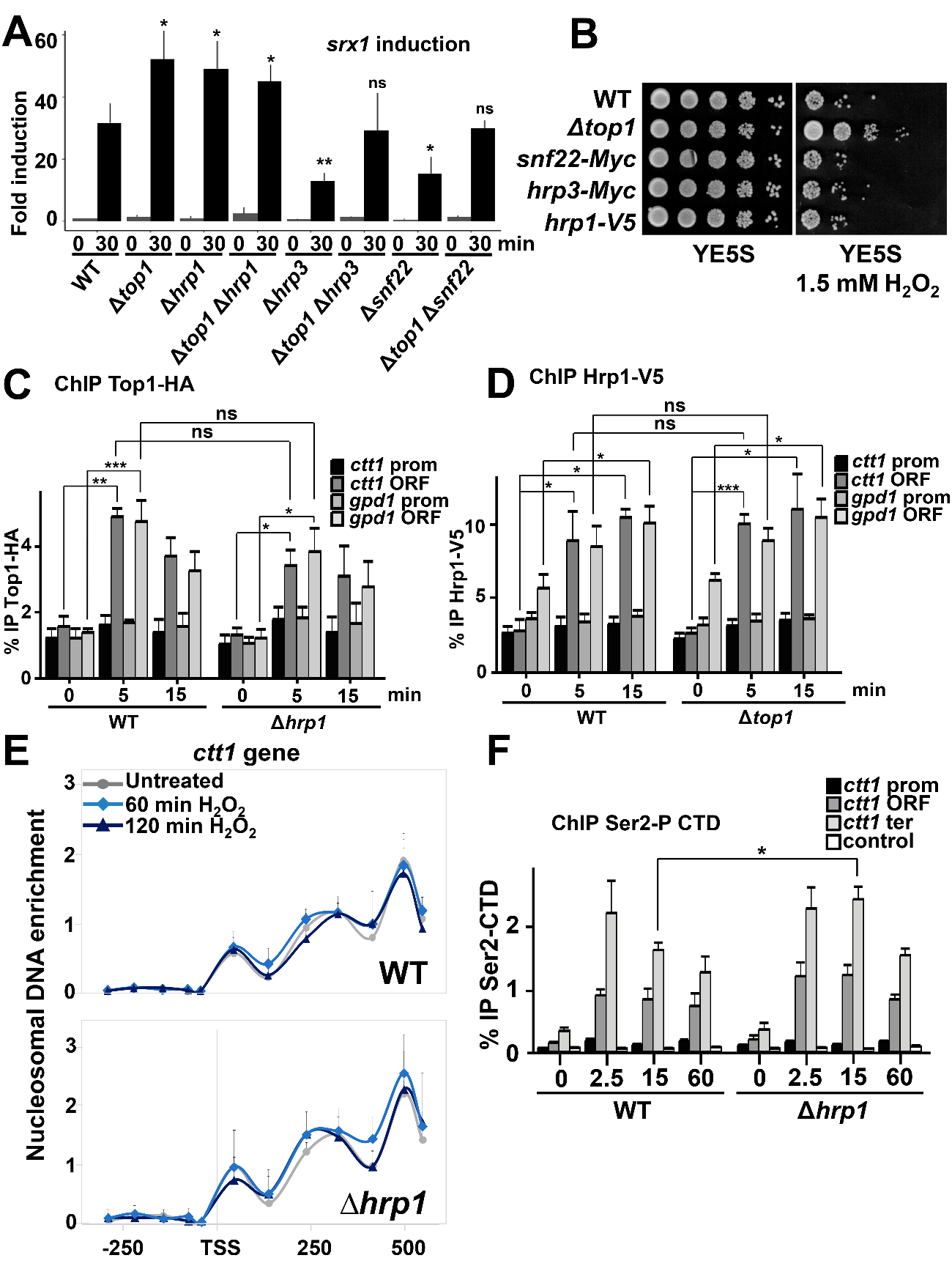


**Supplementary Figure S7.** Characterization of the role of the chromatin remodelers Hrp3, Snf22 and Hrp1 at stress genes. (**A**) Expression of *srx1* was analyzed by quantitative PCR in the indicated strains as explained in Figure 6C. (**B**) Experiments were performed as in Figure 1A using the strains 972 (WT), RF14 (*Δtop1*), JA2653 (*snf22-myc*), JA2777 (*hrp3-myc*) and RB175 (*hrp1-V5*). (**C**) Top1-HA recruitment to stress genes in the presence or absence of Hrp1. ChIP experiments as in Figure 1D using anti-HA antibodies were performed from cultures of strains RF92 (*top1-HA*) and RF100 (*Δhrp1 top1-HA*), using primers covering promoter or ORF regions of *ctt1* and *gpd1*. Primers of a *mtDNA* region were used as control. Bars represent the mean value after normalization to control primers (*mtDNA)* in each replicate, and SD calculated from biological triplicates. Significant differences were determined by a two-sided t-test (*p<0.05, **p<0.01, ***p<0.001, ns, non-significant). (**D**) Hrp1 recruitment to stress genes in the presence or absence of Top1. ChIP experiments were performed as in Figure 1D from strains RB175 (*hrp1-V5*) and RB176 (*Δtop1 hrp1-V5*) using anti-V5 antibody. Bars represent the mean value after normalization to control primers (*mtDNA)* in each replicate, and SD calculated from three biological replicates. Significant differences were determined by a two-sided t-test (*p<0.05, ***p<0.001, ns, non-significant). (**E**) Nucleosome scanning of the *ctt1* gene after long H_2_O_2_ treatments. Nucleosome scanning performed as in Figure 7A-B using 972 (WT) and IV69 (Δ*hrp1*). Lines represent average of biological triplicates with error bars (SD) for untreated conditions (in grey, circles), 60 min (blue, diamonds) and 120 min (dark blue, triangles) of 1 mM H_2_O_2_ stress. (**F**) Recruitment of Pol II phosphorylated at Ser2 of its CTD. ChIP experiments as in Figure 1D using anti-Ser2-P antibodies were performed from cultures of strains 972 (WT) and IV69 (Δ*hrp1*), using primers covering promoter, ORF or terminator regions of *ctt1* gene. Primers of a *mtDNA* region were used as control. Each column represents the mean value and SD, calculated from three biological replicates. Significant differences were determined by a two-sided t-test (*p<0.05).


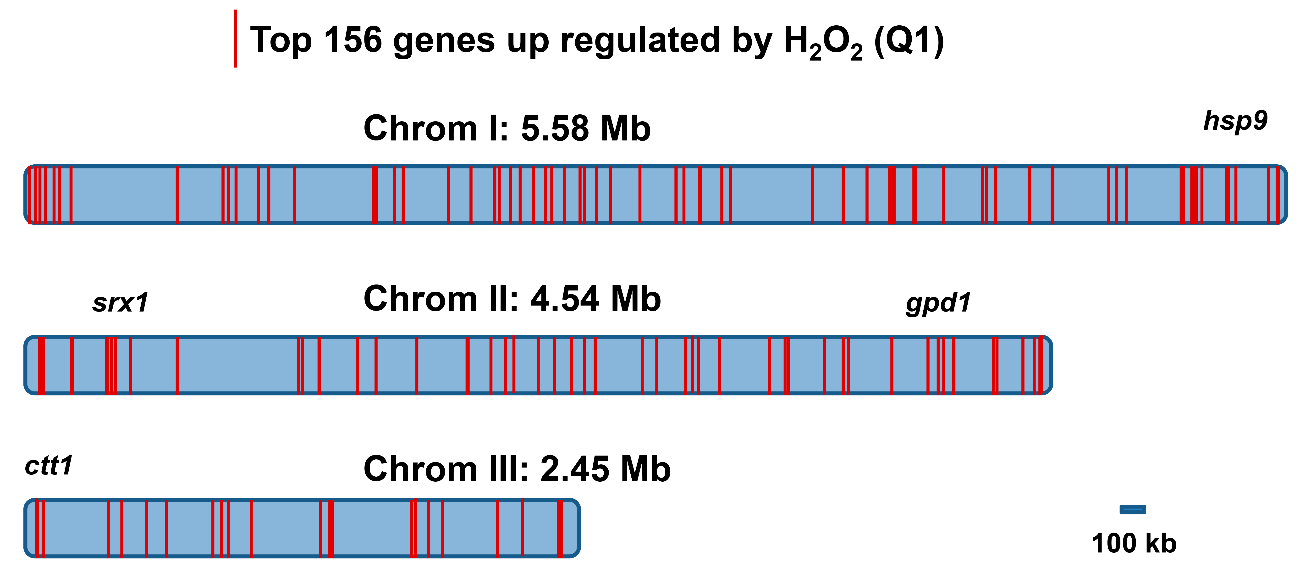


**Supplementary Figure S8.**  Relative chromosomal location of H_2_O_2_ induced genes. Position of the 156 most expressed genes after H_2_O_2_ treatment within the 1-3 *S. pombe* chromosomes. Blue lines represent each of the three *S. pombe* chromosomes and red lines indicate the position of each of the up-regulated genes.

**Table S1.** Strains used in this study

| Strain | Genotype | Origin |
| --- | --- | --- |
| 972 | *h^-^* | ([1](#_ENREF_1)) |
| RF14 | *h^-^ top1::kanMX6* | This work |
| CS38 | *h- atf1-HA::natMX6* | ([2](#_ENREF_2)) |
| RF76 | *h^?^ atf1-HA::natMX6 top1::kanMX6* | This work |
| MS98 | *h^-^ atf1::natMX6* | Lab stock |
| RF52 | *h^?^ atf1::natMX6 top1::kanMX6* | This work |
| RF92 | *h^-^ top1-HA::natMX6* | This work |
| IV69 | *h^-^ hrp1::kanMX6* | This work |
| IV83 | *h^+^ hrp3::natMX6* | This work |
| IV84 | *h^+^ snf22::natMX6* | This work |
| RF99 | *h^?^ hrp1::kanMX6 top1::natMX6* | This work |
| RF117 | *h^?^ hrp3::natMX6 top1::kanMX6* | This work |
| RF118 | *h^?^ snf22::natMX6 top1::kanMX6* | This work |
| RF56 | *h^-^  top1::ura4* | This work |
| RF119 | *h^?^ top1::natMX6 tdp1::kanMX6* | This work |
| RF120 | *h^?^ tdp1::kanMX6* | This work |
| JF5 | *h^-^ rpb3-HA::natMX6* | ([2](#_ENREF_2)) |
| RF96 | *h^?^ top1::kanMX6 rpb3-HA::natMX6* | This work |
| MV118 | *h^-^ top1.Y773F* | This work |
| RB174  RB176 | *h^?^ top1.Y773F-HA::kanMX6*  *h^-^ top1::natMX6 hrp1-V5::kanMX6* | This work  This work |
| EP198 | *h^+^ ctt1::natMX6* | This work |
| RF120 | *h? top1::natMX6 tdp1::kanMX6* | This work |
| JA2653 | *h^-^ snf22-myc::kanMX6* | ([3](#_ENREF_3)) |
| JA2777 | *h^+^ hrp3-myc::kanMX6* | This work |
| RB175 | *h^-^ hrp1-V5::kanMX6* | This work |
| RF100 | *h? hrp1::kanMX6 top1-HA::natMX6* | This work |
| MV117 | *h^?^ ctt1::natMX6 top1::kanMX6* | This work |
| PG102 | *h+ ctt1-GFP::natMX6* | ([4](#_ENREF_4)) |
| MV119 | *h^?^ ctt1-GFP::natMX6 top1::kanMX6* | This work |

| **Table S2.** Primers used in this study for RT-qPCR, ChIP, nucleosome scanning and CRISP-Cas9 experiments | | | |
| --- | --- | --- | --- |
| **Primer** | **Sequence** | **Gene^a^** | **Application** |
| OLEH-H48 | 5´-TGCCCCTAGAGCTGTATTCC-3´ | *act1 F* (cont.) F | qPCR |
| OLEH-H49 | 5´-TTTGAGCTTCATCACCAACG-3´- | *act1 R* (cont.) F | qPCR |
| OLEH-H50 | 5´-CTCGTGTGGGTAAGGGAGGT-3´ | *ctt1* F | qPCR |
| OLEH-H51 | 5´-ATGGACGACACGTTCAGGA-3´ | *ctt1* R | qPCR |
| OLEH-H52 | 5´-CAAGGCCAAGGAATCCATTA-3´ | *hsp9* F | qPCR |
| OLEH-H53 | 5´-GAGCCTTGTCATGAGCCTCT-3´ | *hsp9* R | qPCR |
| OLEH-H56 | 5´-GCTCCGGTAACTGGGGTACT-3´ | *gpd1* F | qPCR |
| OLEH-H57 | 5´-CTCCTCAAAGACCCACATGC-3´ | *gpd1* R | qPCR |
| OLEH-H58 | 5´-TTCATGCGGTTTGACTTCAG-3´ | *srx1* F | qPCR |
| OLEH-H59 | 5´-CCCCCAAAGGCAAAATAATA-3´ | *srx1* R | qPCR |
| OLEH-550 | 5´-TGGAAATAGCTTACGTAGCTCAGAA-3´ | *hsp9* prom F | ChIP |
| OLEH-551 | 5´-ATAGCTCGCTATCCAATCAGACA-3´ | *hsp9* prom R | ChIP |
| OLEH-548 | 5´-TTACGTAATTTTTCCTCTTTGCTTC-3´ | *gpd1* prom F | ChIP |
| OLEH-549 | 5´-TGACGTAAAAACACTACACTTGGTC-3´ | *gpd1* prom R | ChIP |
| OLEH-542 | 5’-GTATTCAACGAAGCTCACGAGAATG-3’ | *gpd1* ORF F | ChIP |
| OLEH-543 | 5’-GCTGACAGCAACACCCTTGATACAG-3’ | *gpd1* ORF R | ChIP |
| OLEH-544 | 5’-CGTCCTTCAACCCCTTAAGGAGGAC-3’ | *gpd1* ter F | ChIP |
| OLEH-545 | 5’-CACACATACAAATAGATACACATGC-3’ | *gpd1* ter R | ChIP |
| OLEH-552 | 5´-TACTAAGAACACAAACCCAGACCTC-3´ | *srx1* prom F | ChIP |
| OLEH-553 | 5´-TGTAAAAATTTGTCAGCATGTTAGC-3´ | *srx1* prom R | ChIP |
| OLEH-923 | 5´-ATAATGATGCTCTTTGGCTCACTAA-3´ | *ctt1* prom F | ChIP |
| OLEH-924 | 5´-CTGGTGTAGAATTACCAACGTCATA-3´ | *ctt1* prom R | ChIP |
| OLEH-925 | 5’-ATCGCTACAACATTTTCGATCTTAC-3’ | *ctt1* ORF F | ChIP |
| OLEH-926 | 5’-AATTGGTAGGATTCTGATTCAAGGT-3’ | *ctt1* ORF R | ChIP |
| OLEH-927 | 5’-CAAGTCTTCTGCCGTAAATTTCTTA-3’ | *ctt1* ter F | ChIP |
| OLEH-928 | 5’-CTCATGAATTTGCTTTTAAGCTTTC-3’ | *ctt1* ter R | ChIP |
| JA488 | 5'-TTTGTCAAAAAGTTTTCCCACATAC-3' | Intergenic (cont.) | ChIP |
| JA489 | 5'-GCGGTTTCCCTTTCTAAGAGACT-3' | Intergenic (cont.) | ChIP |
| JA2026 | 5'-TGGTAGAGCAAGTCACTGTTAATGA-3' | *mtDNA* (cont.) | ChIP |
| JA2027 | 5'-CGTTATAACCGAGCTAACATCAAAC-3' | *mtDNA* (cont.) | ChIP |
| OLEH-B48 | 5´-TTTCATGTCAGCGTCTGTGTC-3´ | *ctt1* F | Nun. scann. |
| OLEH-B49 | 5´-GCCATATCAAATCTCCCAACA-3´ | *ctt1* R | Nun. scann. |
| OLEH-B50 | 5´-TAGCGATGGTGTTGGGAGAT-3´ | *ctt1* F | Nun. scann. |
| OLEH-B51 | 5´-AGCCTATCGTAGTCGCCAAA-3´ | *ctt1* R | Nun. scann. |
| OLEH-B52 | 5´-TGGCGACTACGATAGGCTGT-3´ | *ctt1* F | Nun. scann. |
| OLEH-B53 | 5´-CCGAGATTCCATTGGCTTAG-3´ | *ctt1* R | Nun. scann. |
| OLEH-B54 | 5´-GGCTCACTAAGCCAATGGAA-3´ | *ctt1* F | Nun. scann. |
| OLEH-B55 | 5´-ATTGTCTGGTGTAGAATTACCAACG-3´ | *ctt1* R | Nun. scann. |
| OLEH-B56 | 5´-GCCATTTGCGTTGGTAAGAG-3´ | *ctt1* F | Nun. scann. |
| OLEH-B57 | 5´-TGAAAAGCTGTATATTCAAGCAAC-3´ | *ctt1* R | Nun. scann. |
| OLEH-B58 | 5´-TGCTTGAATATACAGCTTTTCACG-3´ | *ctt1* F | Nun. scann. |
| OLEH-B59 | 5´-TTCGATATTCGAACAAGGGAAT-3´ | *ctt1* R | Nun. scann. |
| OLEH-B60 | 5´-GTAATTCCCTTGTTCGAATATCG-3´ | *ctt1* F | Nun. scann. |
| OLEH-B61 | 5´-GAGGGGAAAAAAAAAGACAGTC-3´ | *ctt1* R | Nun. scann. |
| OLEH-B62 | 5´-TTTTTCCCCTCCTAACTCTTCA-3´ | *ctt1* F | Nun. scann. |
| OLEH-B63 | 5´-GTTGAACGACGAGCAAATGA-3´ | *ctt1* R | Nun. scann. |
| OLEH-B64 | 5´-AATCATTTGCTCGTCGTTCA-3´ | *ctt1* F | Nun. scann. |
| OLEH-B65 | 5´-TCGAGTTCATTTCGCTGATG-3´ | *ctt1* R | Nun. scann. |
| OLEH-B66 | 5´-TCAGCGAAATGAACTCGAAA-3´ | *ctt1* F | Nun. scann. |
| OLEH-B67 | 5´-AGGACCTCCCTTACCCACAC-3´ | *ctt1* R | Nun. scann. |
| OLEH-B68 | 5´-GTGGGTAAGGGAGGTCCTGT-3´ | *ctt1* F | Nun. scann. |
| OLEH-B69 | 5´-ACGACACGTTCAGGAATACG-3´ | *ctt1* R | Nun. scann. |
| OLEH-B92 | 5´-CGTCTTTCAACACTTTGATCG-3´ | *ctt1* F | Nun. scann |
| OLEH-B93 | 5´-GTAATGTCGTCGGTGCATTC-3´ | *ctt1* R | Nun. scann |
| OLEH-G13 | 5´- TGCATCATCTTGGATGCAG-3´ | *ade6* F (control) | Nun. scann |
| OLEH-G14 | 5´-AATTGCATCGGGGTCAGTAA-3´ | *ade6* R (control) | Nun. scann |
| OLEH-AC41 | 5´-GACTAGATTAACTACATAGACCCG-3´ | *top1* sgRNA F | CRISPR-Cas9 |
| OLEH-AC42 | 5´-AAACCGGGTCTATGTAGTTAATCT-3´ | *top1* sgRNA R | CRISPR-Cas9 |
| OLEH-AC43 | 5´-GAACGAATTAATGTTATGCGTACTCAGATGATCGA  TAAAGACGAGAATAAAACTACTGCTTTGGGTACAAGT  AAGATTAACTTCATAGACCCGAGACTTA-3´ | *top1-*HR template F | CRISPR-Cas9 |
| OLEH-AC44 | 5´-AGCCCAATTGAACTTGTCACGAATCGTCTTACTA  AACAGCTTCTCAATAGGAACGTCTTCTCGCTTGCTGA  ACGAATAAGTAAGTCTCGGGTCTATGAAG-3´ | *top1-*HR template R | CRISPR-Cas9 |

**REFERENCES**

1. Leupold, U. (1970) Genetical methods for *Schizosaccharomyces pombe*. *Methods Cell Physiol.*, **4**, 169-177.

2. Salat-Canela, C., Paulo, E., Sanchez-Mir, L., Carmona, M., Ayte, J., Oliva, B. and Hidalgo, E. (2017) Deciphering the role of the signal- and Sty1 kinase-dependent phosphorylation of the stress-responsive transcription factor Atf1 on gene activation. *J Biol Chem*, **292**, 13635-13644.

3. Gonzalez-Medina, A., Pazo, E., Hidalgo, E. and Ayte, J. (2021) SWI/SNF and RSC remodeler complexes bind to MBF-dependent genes. *Cell Cycle*, **20**, 2652-2661.

4. Garcia, P., Encinar Del Dedo, J., Ayte, J. and Hidalgo, E. (2016) Genome-wide Screening of Regulators of Catalase Expression: ROLE OF A TRANSCRIPTION COMPLEX AND HISTONE AND tRNA MODIFICATION COMPLEXES ON ADAPTATION TO STRESS. *J Biol Chem*, **291**, 790-799.
